# Evolution and Impact of Nucleic Acid Amplification Test (NAAT) for Diagnosis of Coronavirus Disease

**DOI:** 10.1021/acs.analchem.3c05225

**Published:** 2024-04-30

**Authors:** Sumbul Fatma Khan, Priyanka Rathod, Vivek K. Gupta, Pramod B. Khedekar, Rupesh V. Chikhale

**Affiliations:** † Department of Pharmaceutical Sciences, Rashtrasant Tukadoji Maharaj Nagpur University, Nagpur 440033, MS India; ‡ Department of Biochemistry, National JALMA Institute for Leprosy & Other Mycobacterial Diseases (ICMR), Agra -282004, India; § UCL School of Pharmacy, Department of Pharmaceutical and Biological Chemistry, University College London, London WC1N 1AX, United Kingdom

## Introduction

**Figure 1:**
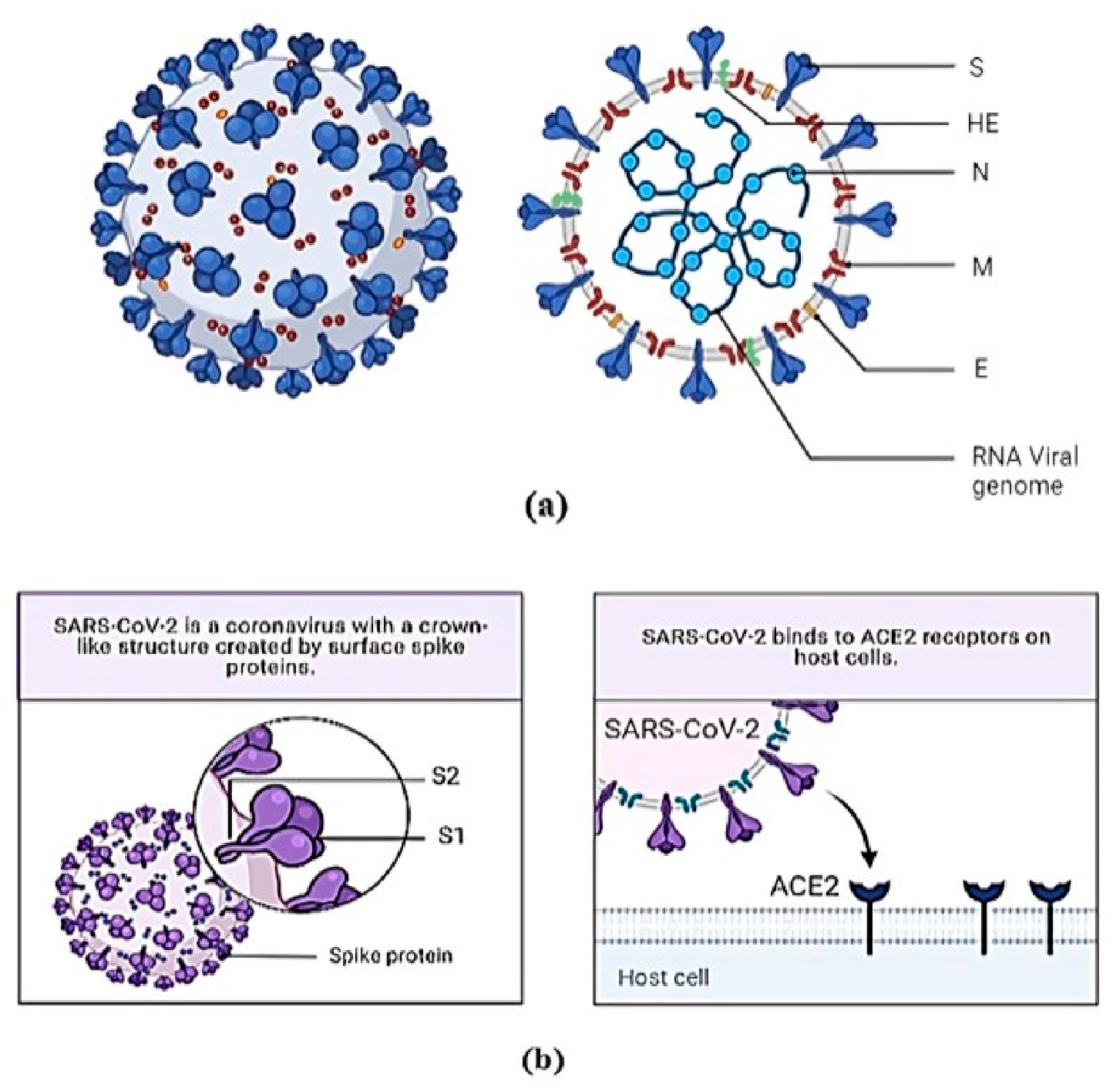
Schematic representation of (a) SARS-Cov-2 virion and its structural protein (b) spike protein denoting S1 and S2 subunit and its binding with ACE2 receptor. [Created with BioRender.com.]

Severe acute respiratory syndrome Coronavirus 2, or SARS-CoV-2, previously known as 2019-CoV, is an encapsulated, positive-sense, single-stranded genomic RNA virus (+ssRNA) that is responsible for causing coronavirus disease 2019 (COVID-19).^1^ New cases of this atypical pneumonia were first reported in Wuhan, Hubei Province, China, in December 2019. The pathogen of this infectious outburst, SARS-CoV-2, affects the lower respiratory tract and some other tissues that possess the ACE2 receptor.^2^ It is highly contagious in humans and has quickly spread worldwide through intimate contact or the release of respiratory secretions (cough, sneeze) from infected individuals. The COVID-19 outbreak was referred to as “a pandemic” by the Director-General of the World Health Organisation (WHO) on March 12, 2020. SARS-CoV-2 is a member of the Coronaviridae family and the *Sarbecovirus* subgenus, including several other viruses that can infect humans in a mild to severe way.^3^ The nucleotide sequence of SARS-CoV-2 is 88% similar to two SARS-like coronaviruses that are generated from bats, known as bat-SL-CoVZC45 and bat-SL-CoVZXC2, 79% identical to SARS-CoV, and 50% similar to MERS-CoV.^4^ The SARS-CoV-2 virus is an enclosed RNA (30 kb) virus that contains a spike protein (S), hemagglutinin-esterase dimer (HE), membrane glycoprotein (M), nucleocapsid protein (N), and an envelope protein (E),^5^ which is shown in Figure 1a. The receptor-binding region of spike protein S attaches to ACE2 (Angiotensin-Converting Enzyme 2), which is characterized as a receptor inside the human host^6^ (Figure 1b). The spike protein, composed of 1273 amino acid residues, consists of two components (S1 and S2) on the virus’s surface. It is highly immunogenic, promotes cell entrance, and targets circulating antibodies.^5^ The S1 subunit helps in the attachment of the virus to the host cell membrane, while the S2 subunit helps in the fusion of virion with the cell membrane. The assembly and release of the virion are the responsibility of the 75 amino acid envelope protein E.^7^ Membrane proteins (M) are 222 amino-acid-long structural proteins that play an essential role in NA packaging and give the virus a definite shape.^8^ Nucleoproteins N are proteins that aid in packaging RNA into ribonucleocapsids.^9^

### Genome Structure of SARS-CoV-2

**Figure 2:**
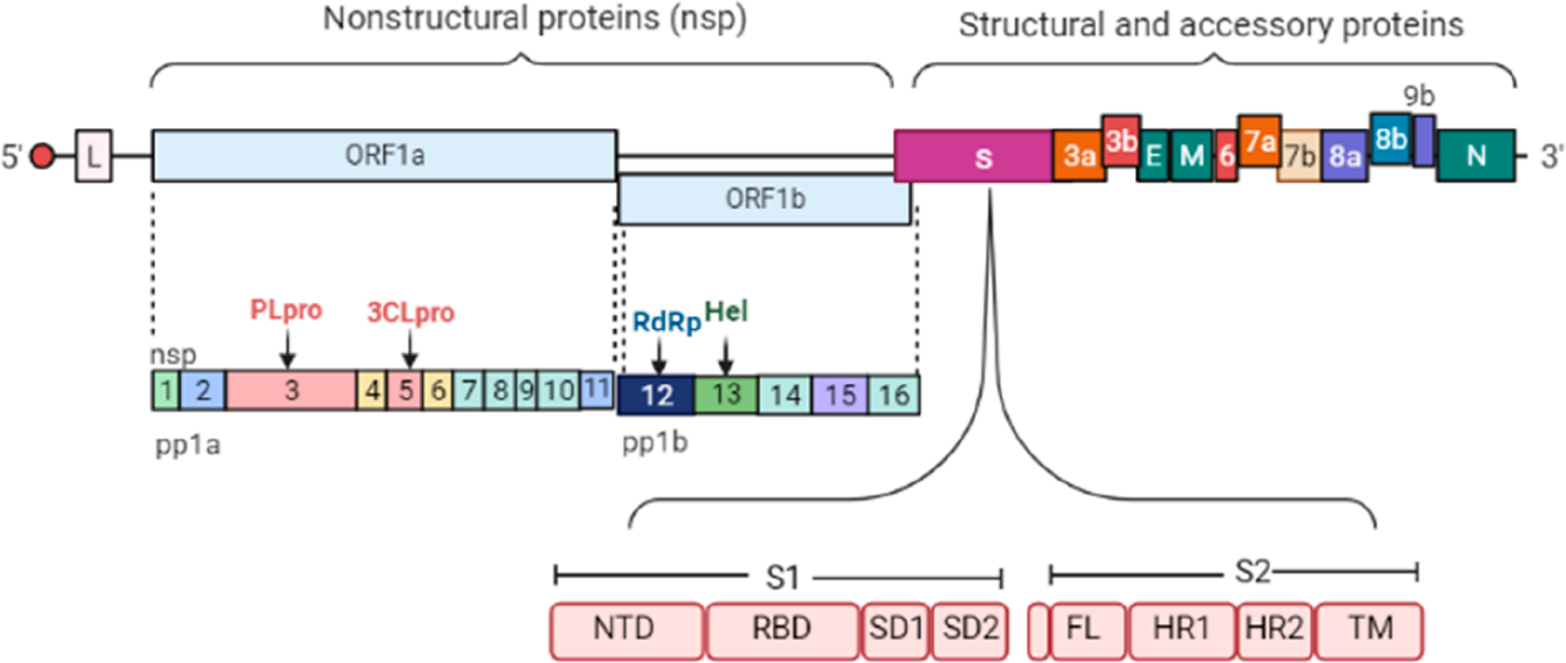
Schematic representation of the SARS-CoV-2 genome, which includes a single-stranded RNA having various Open Reading Frames (ORF1a, ORF1b, ORF3a, ORF3b, ORF6, ORF7a, ORF7b, ORF8a, ORF8b, ORF9b), leading to the formation of 16 nonstructural protein (nsps1–nsps16), structural proteins like S (spike protein) having the S1 subunit, which includes NTD, RBD, SD1, and SD2 and the S2 subunit, which contains FL, HR1, HR2, and TM. Other structural proteins are E (Envelop protein), M (Membrane protein), N (Nucleocapsid protein). PL-pro (papain-like protease), and 3CL-pro (cysteine proteases). [Created with BioRender.com.]

The genome of SARS-CoV-2 is composed of a single-stranded positive-sense RNA (Figure 2). According to recent reports, the genome size of SARS-CoV-2 was sequenced as 29.9 kb.^10^ Other than the structural proteins (E, M, N, S), the genome includes various open reading frames (ORFs). The first ORF has two units comprising 67% of the genome. The ORF1a and ORF1b are translated to Polyprotein 1a and 1b, respectively. The virus genome encodes two cysteine proteases: a papain-like protease (PLpro) or Non-Structural protein 3 (nsp3) and a 3C-like protease (3CLpro) or nsp5. These proteases slice pp1a and pp1b polypeptides into 16 nonstructural proteins.^11^ The ORF1a encodes 1–11 nonstructural proteins (nsps), whereas ORF1b encodes 12–16 nsps. Out of 16 nsps, nsps12, i.e., RNA-Dependent RNA Polymerase (RdRp), is the primary target for the antiviruses, since it is responsible for replication/transcription.^12^ After ORF1a and ORF1b, there is protein S, which consists of two subunits: S1 and S2. S1 consists of an N-terminal domain (NTD), Receptor Binding Domain (RBD) or C-terminal Domain 1 (CTD1), Subdomain 1 (SD1) and Subdomain 2 (SD2) or CTD-2, while S2 comprises Fusion Loop (FL), Heptad Repeat (HR1), and HR2 and Transmembrane Domain (TM).^13^ The domains of the S1 subunit are mainly responsible for target recognition and attachment. The role of NTD is still undiscovered but is responsible for the structural conformation of protein S.^14^ NTD facilitates the infection in other related coronaviruses by recognizing glycoproteins.^15^ The receptor binding domain is responsible for membrane fusion of the virus particle. The S2 subunit contains a fusion loop, which is critical in fusogenicity. The fusion loop spatially arranges three consecutive fusion peptides to stimulate the virus-host membrane fusion process^16^ effectively. HR1 and HR2 interact with each other and form a 6-helical bundle, thus bringing the virus nearby, which facilitates the fusion.^16^ The remaining genome consisted of other overlapping ORFs responsible for the translation of structural proteins (E, M, N, S).^17^

### Nucleic Acid Amplification Tests (NAATs)

**Figure 3:**
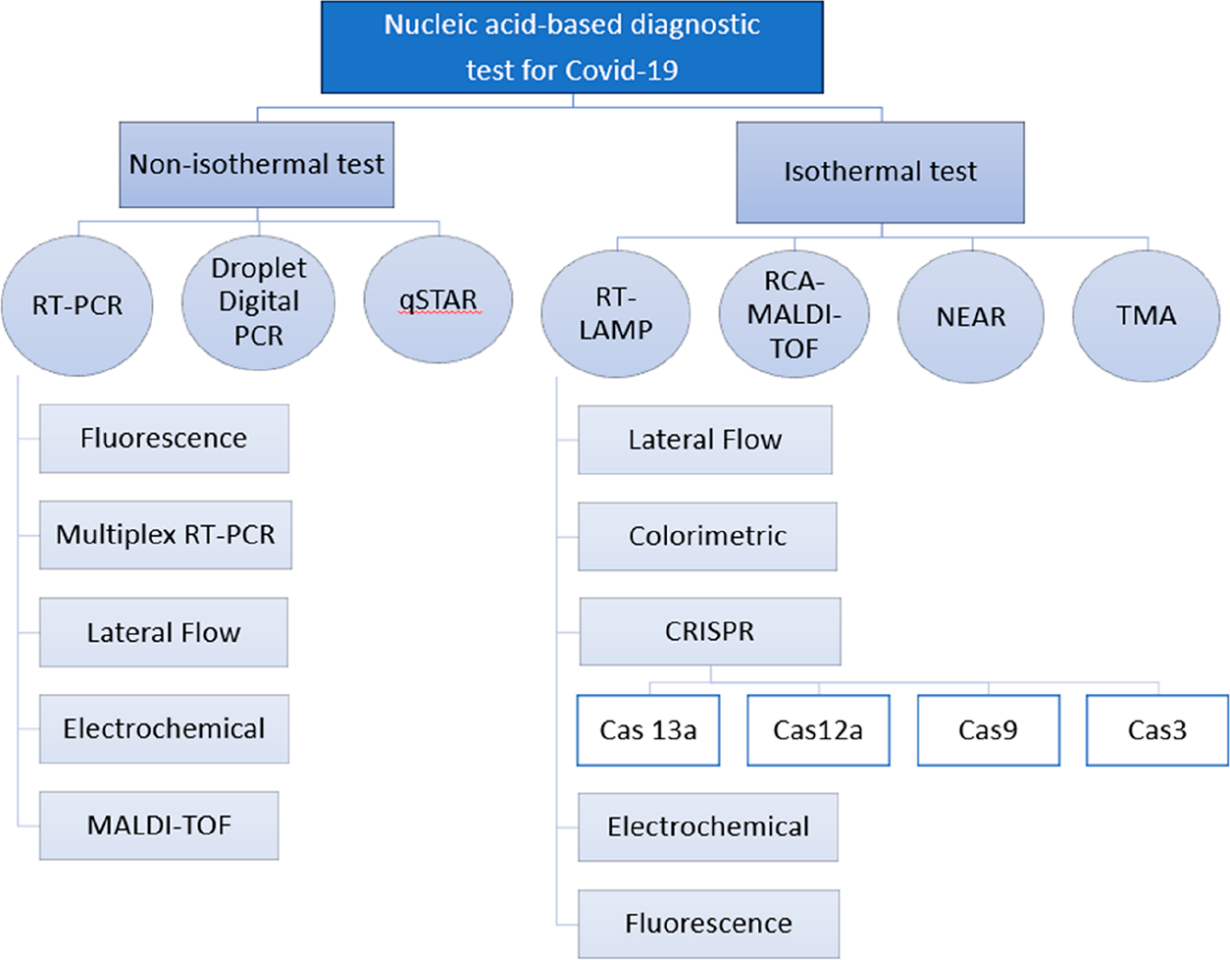
Classification of nucleic-acid-based diagnostic tests for Covid-19.

A type of viral diagnostic test known as a Nucleic Acid Amplification Test (NAAT) is used to amplify the target sequences that constitute the genetic makeup of the virus and then identify them using a read-out detection procedure such as a fluorescent probe assay, a lateral flow assay. The test directly targets SARS-CoV-2 nucleic acid in the nasopharyngeal swab, fluid from bronchoalveolar lavage, saliva, blood, sputum, throat, or anal swab, tissues from a biopsy or autopsy, incorporating lung samples, and urine samples.^28^ The WHO published a guideline that includes NAATs as the primary tests to detect viral RNA in suspected cases (Figure 3). These tests mainly help in the early detection of SARS-CoV-2 (Table 1) and, therefore, have emerged as a practical requirement for controlling the illness, providing timely treatment, and further preventing the spread and severity of the disease.^29^

**Table 1:** Important Genes Targeted for COVID-19 Diagnosis and Their Salient Features

No.		genes	range of amino acids	salient features	ref
1		ORF1a	–	Encodes pp1a, which, after splitting, forms nsp1 nsp11, which are involved in various transcription and replication processes. ORF1a is also a significant target for COVID-19 diagnosis and can be detected by multiple amplification tests.	
1		nsp1	1–180	Incorporates the carboxy terminal domain into the mRNA entry tunnel of the host ribosomal 40S subunit, thus inhibiting the host translation. Interacts with the host cell antiviral machinery.	(18)
1		nsp2	181–818	Interacts with host protein complexes; Prohibitin 1 and 2 are involved in the host cell survival signaling pathway.	(19)
1		nsp3	819–2763	A papain-like protease contains several domains and separates the translated polyproteins into distinct proteins.	(20)
1		nsp4	2764–3263	Membrane-spanning protein anchors the viral replication-transcription complex to endoplasmic reticulum membranes. Plays critical functions during viral RNA synthesis and double-membrane vesicle formation.	(21)
1		nsp5	3264–3569	It splits the large polyproteins into the individual nsps, and it is an essential part of viral replication-transcription complexes.	(21)
1		nsp6	3570–3859	Involved in forming double membrane vesicles, which protects viral RNA from detection and degradation. Modulates the function of autophagy in the host cell.	(21)
1		nsp7	3860–3942	Forms a hexadecameric super complex with nsp8 and serves as a cofactor for nsp12, thus indirectly enhancing the viral replication.	(21)
1		nsp8	3943–4140	Forms a complex with nsp7 and helps in nsp12 processivity.	(21)
1		nsp9	4141–4253	Viral replication, overall virulence, and viral genomic RNA reproduction.	(22)
1		nsp10	4254–4392	With the help of nsp16, it performs the 2′-O-methylation of viral RNA cap structure, which ultimately helps in degrading the host cell immune system.	(21)
1		nsp11	4393–4405	13-amino-acid-long intrinsically disordered protein (IDP). It may play a functional role in the host cytosolic membrane affinity/interaction.	(23)
					
2		ORF 1b	–	They are involved in RNA replication and transcription, perform helicase activity, and proofread and repair viral RNA.	
2		nsp12	4406–5324	Responsible for replication and transcription of the viral RNA genome.	(21)
2		nsp13	5325–5925	RNA helicase, responsible for the unwinding of dsRNA; its zinc-binding domain is involved in replication and transcription.	(21)
2		nsp14	5926–6452	Exhibits exoribonuclease activity responsible for proofreading and repairing viral RNA during replication. Removes the inappropriate nucleotides. It interacts with nsp16 and increases its activity. The C-terminal region causes *S*-adenosyl methionine-dependent guanine-N7 methyl transferase independent of the ExoN activity.	(24)
2		nsp15	6453–6798	A uridine-specific endoribonuclease is responsible for proofreading in viral replication. Cleaves viral RNA in such a way that degrades the fragments of viral RNA, which can trigger the host cell’s immune responses.	(25)
2		nsp16	6799–7096	With the help of nsp10, it performs the 2′-O-methylation of viral RNA cap structure, which ultimately helps in degrading the host cell immune system. 2′-O-methylation also increases the stability of viral RNA. Mimics the human protein Cap-specific mRNA (nucleoside-2′-O−)-methyltransferase (CMTr1) to perform a crucial step in capping transcribed mRNA.	(26)
					
3		S		Spike protein consisting of S1 and S2 domains.	
	**S1**	NTD		Responsible for structural conformation of protein S. In other related coronaviruses, NTD facilitates the infection by recognizing glycoproteins.	(14)
	**S1**	RBD		Responsible for membrane fusion of the virus particle	(14)
	**S2**	FL		Plays a critical role in fusogenicity. Spatially arranges three consecutive fusion peptides to stimulate the virus–host membrane fusion process effectively.	(16)
	**S2**	HR1 and HR2		Interacts with each other and forms a 6-helical Bundle, thus bringing the virus close, facilitating the fusion.	(16)
					
4		E		It interacts with viral M protein and maintains the structure of viral particles. Homo-oligomerizes to form a cation-selective ion channel called viroporin. Viroporin influences the ionic balance of host cells and may release viral particles into it. This process is also known as viral budding.	(27)
					
5		M		Coordinates with protein E and maintains the formation and integration of viral envelopes, also determines the size and curvature of viral envelopes. It interacts with protein N and causes the assembly of new virions.	(21)
					
6		N		Causes the formation of nucleocapsid, essential for protecting viral RNA from degradation. Responsible for viral assembly by interacting with protein M, which is helpful in the augmentation of viral RNA transcription and replication.	(21)

### Difference between Isothermal and Nonisothermal Techniques

Differences between Isothermal and nonisothermal techniques are mentioned in Table 2.

**Table 2:** Difference between Isothermal and Nonisothermal Techniques

isothermal techniques	nonisothermal techniques
Maintains a constant temperature throughout the reaction, ∼60–65 °C	It involves cycling through different temperatures for denaturation, primer annealing, and extension.
**Advantages:**	**Advantages:**
• No need for Complex thermal cycling equipment.	• Highly sensitive and can detect low amounts of target DNA.
• Faster results due to continuous amplification at a constant temperature	• Multiple targets can be amplified in the same reaction with different primers.
	
**Challenges:**	**Challenges:**
• Designing a specific primer can be challenging	• Time-consuming thermal cycling steps.
• Multiplexing multiple targets is complex	• Requires specialized equipment for precise temperature control.
	
**Example:**	**Example:**
• Loop-Mediated Isothermal Amplification (LAMP) technique	• Reverse Transcription–Polymerase Chain Reaction.
• Clustered Regularly Interspaced Short Palindromic Repeats (CRISPR)/Cas-Based Tests	• Selective Temperature Amplification Reaction (qSTAR)
• Rolling circle amplification (RCA) with MALDI-TOF-MS	• Droplet Digital-Reverse Transcription–Polymerase Chain Reaction.
• Nicking Enzyme Amplification Reaction (NEAR)	

### Nonisothermal Techniques

Nonisothermal nucleic acid amplification produces numerous copies of targeted nucleic acid (RNA/DNA) through reiterative cycling at different temperatures.

### RT-PCR

Reverse transcription-PCR, which the WHO has defined as the standard for assessing SARS-CoV-2 illness, is the method most frequently employed for detecting COVID-19 in laboratories. RT-PCR is a PCR-based technique for RNA (genomic) detection. The RT-PCR test is an efficient method that yields results within a few hours with high throughput.

### RT-PCR with Fluorescence Detection

**Figure 4:**
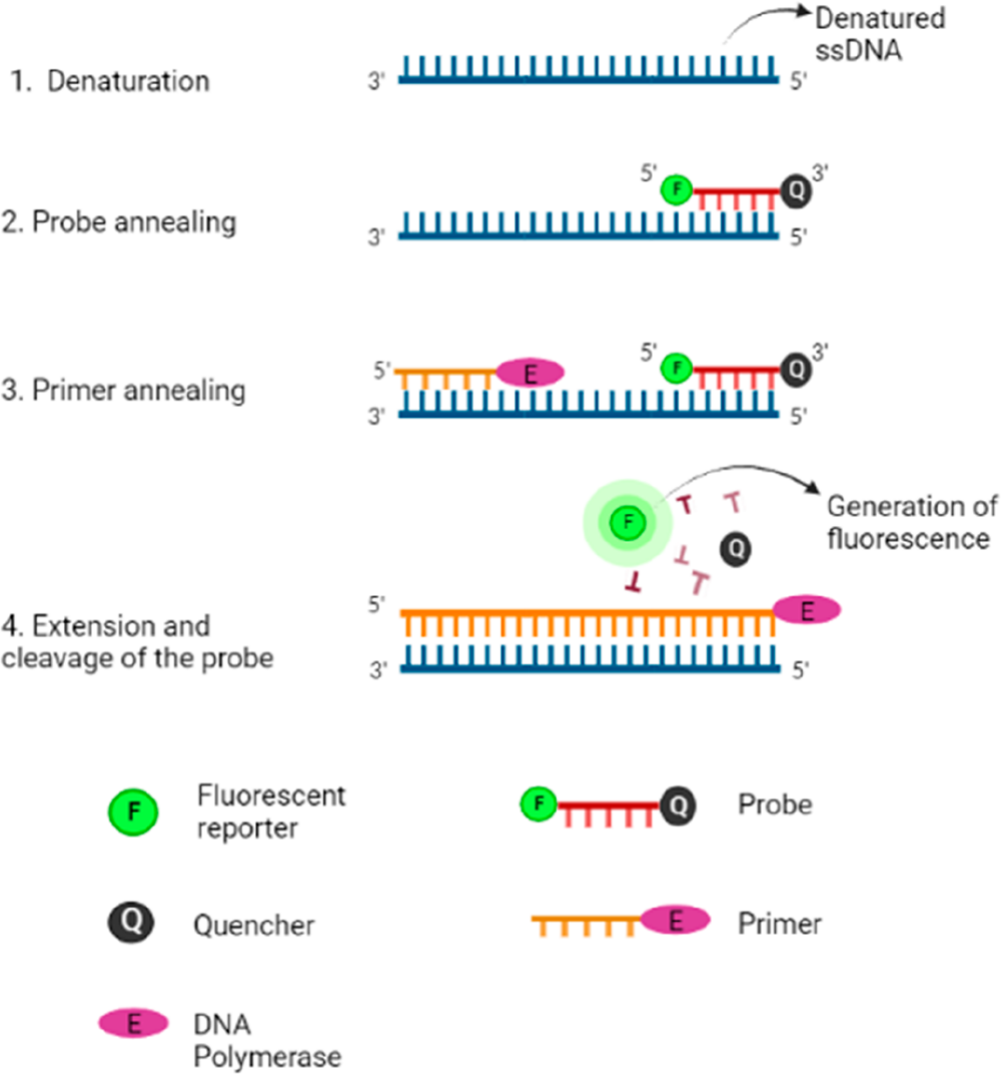
Principle of PCR based on fluorescence detection. 1, Formation of ssDNA. 2, Annealing of the fluorescently labeled probe with ssDNA. 3, Annealing of enzyme-linked primer with ssDNA. 4, Polymerase causes extension of complementary DNA strands, leading to cleavage of the fluorescent probe, which emits fluorescence. [Created with BioRender.com.]

The RT-PCR method’s basic idea is that a reverse transcription enzyme converts RNA into complementary DNA (cDNA). The cDNA is then amplified by a polymerase chain reaction with hydrolyzable fluorescently tagged probes and gene-specific primers (Figure 4).^30^ The probe is used to sense the existence of a particular DNA fragment in the mixture, while the primer is used to initiate the polymerase chain reaction. Denaturation, annealing, and extension are PCR’s three heat cycling phases, represented in Table 3.

**Table 3:** Phases of Polymerase Chain Reaction

phase	temperature (°C)	function
denaturation	95	separation of both the strands of cDNA
annealing	58	forward primer attaches to the complementary site at the ss RNA
extension	72	DNA polymerase synthesizes new DNA strands complementary to the DNA template strand

After the first cycle, the obtained dsDNA again undergoes denaturation to get two ssDNA; when a fluorescent probe is introduced to the reaction mixture, it gets attached to the complementary site situated at one of the ssDNA and a gene-specific primer linked with DNA polymerase, also anneals to its complementary site. The DNA polymerase carries out the extension; as soon as the polymerase reaches the fluorescently labeled probe, it hydrolyzes the probe by separating the fluorescent molecule from the probe. A rise in the fluorescence signal enables the detection of the proportion of duplicate DNA to be done in real time. The number of cycles required for the fluorescent signal to surpass the threshold during the exponential phase of the amplification response is known as the cycle threshold (Ct).^31^ Ct levels are inversely proportional to the sample’s target nucleic acid amount. Ct values <40 are suggested as indicators of SARS-CoV-2 RNA positivity (see Table 4).^32^

**Table 4:** Limits of Cycle Threshold (Ct) To Indicate the Amount of Nucleic Acid Present

Ct value	indication
15–24	the abundant amount of target nucleic acid
25–30	a moderate amount of target nucleic acid
33–40	a minimal amount of target nucleic acid

Among various available probes, well-known TaqMan hydrolysis probes are mainly used for testing, because the fluorophore and quencher are close together in a TaqMan probe. This hydrolysis probe is connected with a fluorescent reporter at the 5′ end and a quencher at the 3′ end. Fluorescein amidites (FAM) emit green fluorescence, and Black Hole Quencher 1 dye is generally employed as fluorescent reporter–quencher pairs. As long as such an oligonucleotide probe remains unbroken, no fluorescence signal is observed. Still, as the probe hydrolyzes in the PCR and the fluorescent reporter–quencher pairs separate, it emits the fluorescent, which is proportional to the amount of particular nucleic acid present.^33^ However, the United States Centers for Disease Control and Prevention (CDC) observed a fluorescence signal without viral nucleic acid (false positive reactivity). The N3 primer and probe were finally removed from the diagnostic kit due to the persistent false-positive findings for the N3 oligonucleotide set but not for N1. This resulted in a limited number of valid tests performed in February 2020 in the United States.^34^ The FDA then granted an Emergency Use Authorization (EUA) in late February 2020 for the use of the CDC’s validated test kit, dubbed “CDC 2019–Novel Coronavirus (2019-nCoV) Real-Time RT-PCR Diagnostic Panel”, to be used in accredited laboratories. The test includes internal control (IC) and the target N gene’s primer-probe sets (N1 and N2) (RNase P). In this test, the viral RNA is extracted and purified from respiratory samples, reverse transcribed to its cDNA, and amplified on the Applied Biosystems 7500 Fast Dx Real-Time PCR Equipment, using SDS version 1.4 software. The probe anneals a precise sequence between the forward and reverse primers during the reaction. In the extension phase of the PCR cycle, the probe is hydrolyzed by Taq polymerase’s 5′ nuclease activity, separating the reporter dye from the quencher dye and generating a fluorescence signal. The fluorescence intensity and the quantity of reporter dye break free from their probes increase. The CDC 2019-nCoV Real-Time RT-PCR Diagnostic Panel has a limit of detection of 10^0^–10^0.5^ copies/ μL, and, thus, the test showed 100% positive agreement (PPA; 95% CI: 77.2%–100%) and 100% negative agreement (NPA; 95% CI: 96.4%–100%).^35,36^

### Multiplex RT-PCR

Multiplex quantitative RT-PCR assay for SARS CoV-2 has been established to detect all variants of concern and mutations in the viral genome. Multiplex real-time RT-PCR detects multiple targets in a single reaction and can simultaneously differentiate from other viruses or microorganisms. Explicitly focusing on all SARS CoV-2 variants of concern (VOC), including the alpha, beta, gamma, delta, and omicron, as well as a significant spike protein mutation, Ryan et al. suggested this multiplex test. They used a molecular beacon instead of a linear probe. A molecular beacon is a single-stranded nucleic acid probe consisting of a loop and a stem attached with a fluorophore and a quencher.^37^ It only binds to targets that vary from the mutant by one nucleotide. The molecular beacon is more targeted than linear oligonucleotides without stem and loop structures. The test has a detection limit of 50 copies/mL and 100% specificity.^38^ The SARS-CoV-2 (Flu SC2) Multiplex Assay,^39^ Cepheid’s Xpert Xpress SARS-CoV-2/Flu/RSV,^40^ and Roche’s Cobas SARS-CoV-2 and Influenza A/B^41^ all can recognize and distinguish between SARS-CoV-2 and Influenza A/B, and the last technique can also detect RSV. QIAstat-Dx Respiratory SARS-CoV-2 Panel (Qiagen) is a programmed diagnostic system that provides a Multiplexed RT-quantitative PCR test to identify 21 different respiratory viruses and bacteria, such as SARS-CoV-2, *Bordetella pertussis*, Chlamydophila pneumonia, Mycoplasma pneumonia, Coronavirus 229 E, Influenza A, Influenza A subtype H1N1/2009, Influenza A subtypes H1, H3, and Influenza B.^42^

### RT-PCR with Lateral Flow

The lateral flow assay (LFA) subjects the sample to a unidirectional flow in a liquid medium and enables the identification of analytes in a test strip. Specific targets can interact more readily with molecules immobilized on a solid surface.^43^ To screen for symptomatic infection and population-wide illness, lateral flow tests (LFTs) are strongly recommended. LFTs do not require a laboratory, so they are substantially more inexpensive, more straightforward to make, and provide findings much more quickly, within 15–30 min on-site.^44^ Once the amplification is complete, the next step is the detection of the amplified DNA. In RT-PCR with lateral flow detection, a lateral flow strip is used as the detection platform. This strip consists of several zones that facilitate the visualization of the amplified DNA.

(1) Conjugate Pad: This zone contains AuNPs coated with specific antibodies or probes complementary to the amplified DNA sequence. The antibodies or probes are conjugated with colored particles, typically red or blue.
(2) Sample Pad: The sample pad is where the amplified DNA and any other necessary reagents are applied.
(3) Test Line: The test line contains immobilized capture probes complementary to the amplified DNA sequence. A visible line appears when the amplified DNA binds to the capture probes.
(4) Control Line: The control line contains immobilized control probes that bind to a different sequence in all samples. It serves as a positive control to verify the proper functioning of the lateral flow strip.
(5) Detection and Interpretation: To perform the lateral flow detection, the amplified DNA sample is applied to the sample pad. The amplified DNA migrates along the strip through capillary action. The AuNPs bind to the DNA as it passes through the conjugate pad, forming a gold–DNA complex.

The gold–DNA complex continues to migrate and reaches the test line. If the amplified DNA sequences in the sample, it will bind to the capture probes on the test line, forming a visible line. This indicates a positive result for the presence of the target sequence. Simultaneously, the gold–DNA complex moves past the test line and reaches the control line. The control probes on the control line bind to the control sequence, regardless of the presence of the target sequence. The control line serves as a verification that the test is functioning correctly. A visible line on the control line confirms the validity of the test. The interpretation of the test results is based on the presence or absence of visible lines on the test and control lines. If both lines are visible, it indicates a positive result, indicating the presence of the target sequence. If only the control line is visible and the test line is absent, it shows a negative result, meaning that the target sequence is absent.

A sample to answer nucleic acid amplification of Accula SARS-CoV-2 was performed within 30 min by (Mesa Biotech, Inc., San Diego, CA). The nucleocapsid protein (N) gene is targeted using reverse transcription-PCR (RT-PCR), and the results are detected using lateral flow. The Accula SARS-CoV-2 Test was carried out on the Accula Dock, or Silaris Dock, which is a palm-sized dock to sustain the reaction temperatures, fluid movements, and timing inside the self-contained test cassette. The nasal or nasal midturbinate samples are introduced to the SARS-CoV-2 buffer to solubilize the sample. This is followed by introducing a portion of the SARS-CoV-2 buffer to an Accula SARS-CoV-2 cassette for amplification. Blue test lines on the detecting strip of the test cassette are used to evaluate the results. A blue process control line is used in the control (C) region, to ensure appropriate reagent and Accula Dock operation and certify that a negative test result is authentic. The limit of detection (LoD) for the test is 200 copies/reaction with PPA and NPA 95.8% (95% CI: 78.9%–99.9%) and 100% (95% CI:86.8%–100%).^45,46^

### RT-PCR with Electrochemical Detection

**Figure 5:**
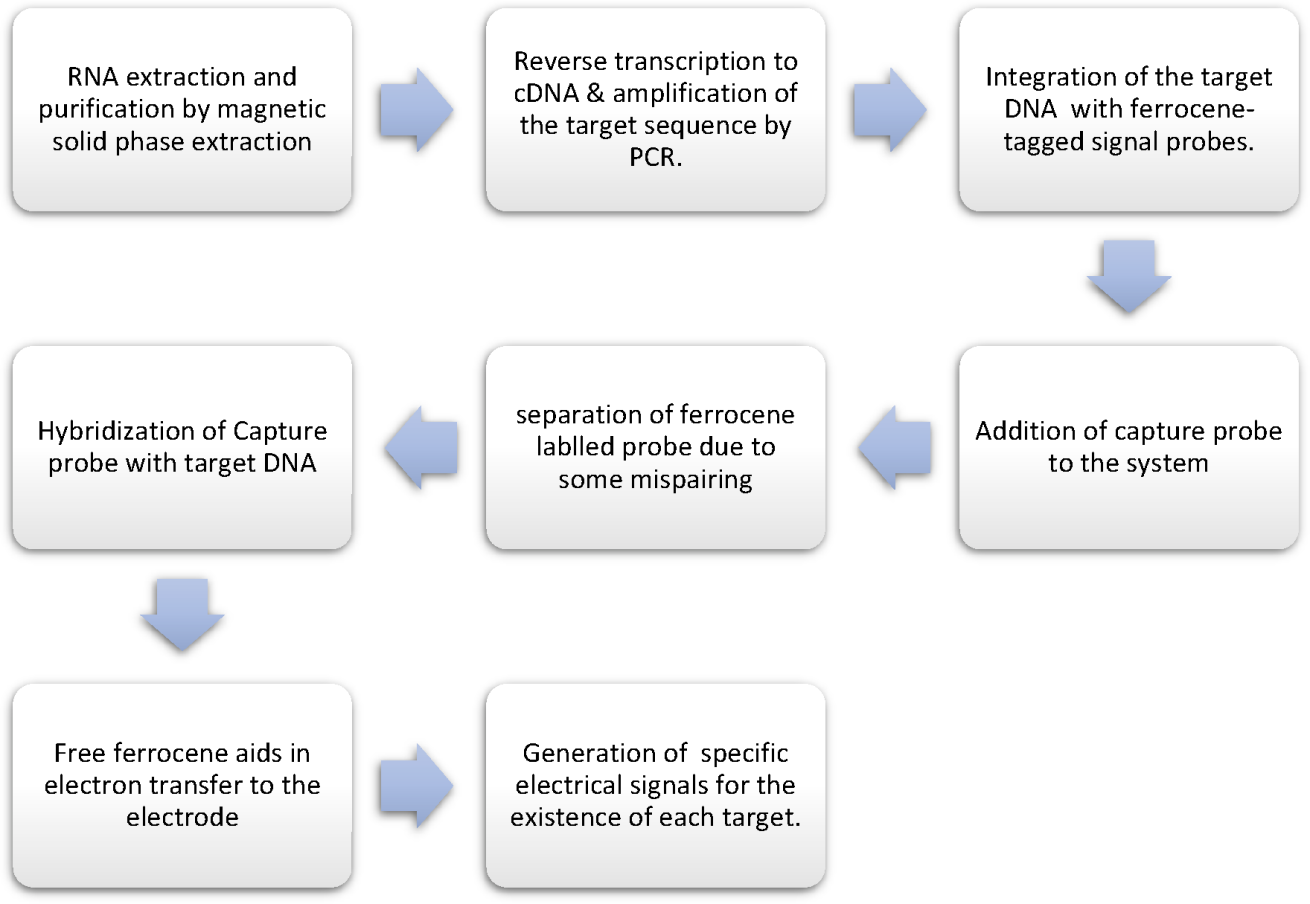
General workflow of RT-PCR with electrochemical detection.

Electrochemical detection methods are sensitive, easy to handle, quick, cost-effective, and require less evaluation time.^47^ ePlex SARS-CoV-2 Test and ePlex Respiratory Pathogen Panel 2 (ePlex RP2 Panel),^48^ established by GenMarkDx, applied e-Sensor technology. Both tests were completed within 2 h. The test contains an ePlex cartridge that completes the sample processing process, from nucleic acid extraction to identification. The workflow of RT-PCR with electrochemical detection is given in Figure 5.^49^ The ePlex SARS-CoV-2 Test detects one viral target, while the ePlex RP2 Panel determines 16 viral and bacterial targets.^50^ The ePlex RP2 Panel characteristically performs better, such as lower LoD (250 genomic copies/mL) and higher PPA (100%, 95% CI: 93.9%–100%) and NPA (100%, 95% CI: 96.7%–100%).^49^ LoD, PPA, and NPA results for the ePlex SARS-CoV-2 Test were 750 genomic copies/mL, 94.4% (95% CI: 74.2%–99.6%), and 100% (95% CI: 92.4%–100%), respectively.^51^

### RT-PCR with MALDI-TOF Detection

MALDI-TOF mass spectroscopy technique in virology identifies several mutations in viruses and also detects various strains, which aids in the quick and precise diagnosis of viral infection.^52^ MALDI-TOF MS technology has the potential to generate mass spectra from nasopharyngeal swabs. Specific selective peaks can be found and used to differentiate positive and negative samples of COVID-19.^53^ SARS-CoV-2 MassArray Test and Ethos Laboratories SARS-CoV-2 MALDI-TOF Assay^54,55^ employed the Agena SARS-CoV-2 Panel test kit for the amplification and detection of SARS-CoV-2 RNA. The RNA is extracted from saliva in the Agena SARS-CoV-2 Panel and is subjected to RT-PCR in IPLEX Pro Chemistry. Then, five sets of PCR primers were used to amplify the genome’s DNA target, two sections of the ORF1ab gene, and three of the nucleocapsid gene (N1, N2, N3). After this, Short Amplicon Primer (SAP) treatments were employed to remove the phosphate group from any remaining free deoxynucleotides to avoid extension interference. In the extension stage, the primers were extended using one of the terminator nucleotides—A, T, C, or G—resulting in allele-specific extension products of various masses. Desalted extension products (analytes) were put into a MALDI-TOF mass spectrometer (MassARRAY Analyzer) using a silicon chip with a prespotted matrix crystal (SpectroCHIP Array). The analyte/matrix co-crystals were exposed to a laser, which caused desorption and ionization. Positively charged molecules were propelled into a flight tube and toward a detector. Time of flight, which is inversely related to molecular mass, separates the molecules. After data processing, each analyte received a spectral fingerprint that describes the molecules’ mass/charge ratio and relative intensity.^54^ The MassARRAY Analyzer data were analyzed using the MassARRAY Typer software and the SARS-CoV-2 Report software. The process takes 8.3 h, including 28 min of hands-on time, from RT-PCR amplification through MassARRAY result production. Different LoDs have been observed for the SARS-CoV-2 MassArray Test (0.69–2.75 copies/mL), Agena SARS-CoV-2 Panel (2.5 copies/mL), and Ethos Laboratories SARS-CoV-2 MALDI-TOF Assay (1 TCID50/mL), with PPA and NPA values for the three tests ranging from 95% to 100%. This has been attributed to differences in RNA extraction kits and equipment used.

### Selective Temperature Amplification Reaction (qSTAR)

qSTAR technology (Selective Temperature Amplification Reaction) is utilized by both LumiraDx SARS-CoV-2 RNA STAR and LumiraDx SARS-CoV-2 RNA STAR Complete. LumiraDx SARS-CoV-2 RNA STAR eliminates nucleic acid extraction and directly administers the specimen into the qSTAR reaction mixture with extraction buffer. The detergent in the 10× extraction buffer lyses the SARS-CoV-2 virion in the LumiraDx SARS-CoV-2 RNA STAR Complete assay. Hence, the total assay time is reduced by merging the extraction and amplification steps into one. Before being amplified by qSTAR, the RNA is reverse-transcribed to produce cDNA. When cDNA is amplified using qSTAR, the polymerase activity is typically favored at a higher temperature (61 °C), but the nicking enzyme activity is frequently desired at a lower temperature (54 °C). A nicking site is built into the primers at both ends of the duplex amplicon. The polymerase displaces the downstream nontemplate strand while extending the nicked primer to create a new strand, restoring the nicking site. This happens after the nicking enzyme forms a single-stranded nick in the duplex. The target amplicons are produced in many copies due to temperature switching. Unlike fluorophores (FAM and ROX), molecular beacons are responsible for the real-time detection of the ORF1a and IC amplicons. qSTAR enzymes can amplify nucleic acid within 5 min, whereas PCR generally takes over an hour. The tests do not employ a Ct cutoff in the analysis process; instead, they provide the probable Ct value for the real-time method. In comparison to LumiraDx SARS-CoV-2 RNA STAR Complete (LoD = 7500 copies/mL), LumiraDx SARS-CoV-2 RNA STAR has a lower LoD (500 copies/mL).^55^

### Droplet Digital RT-PCR

**Figure 6:**
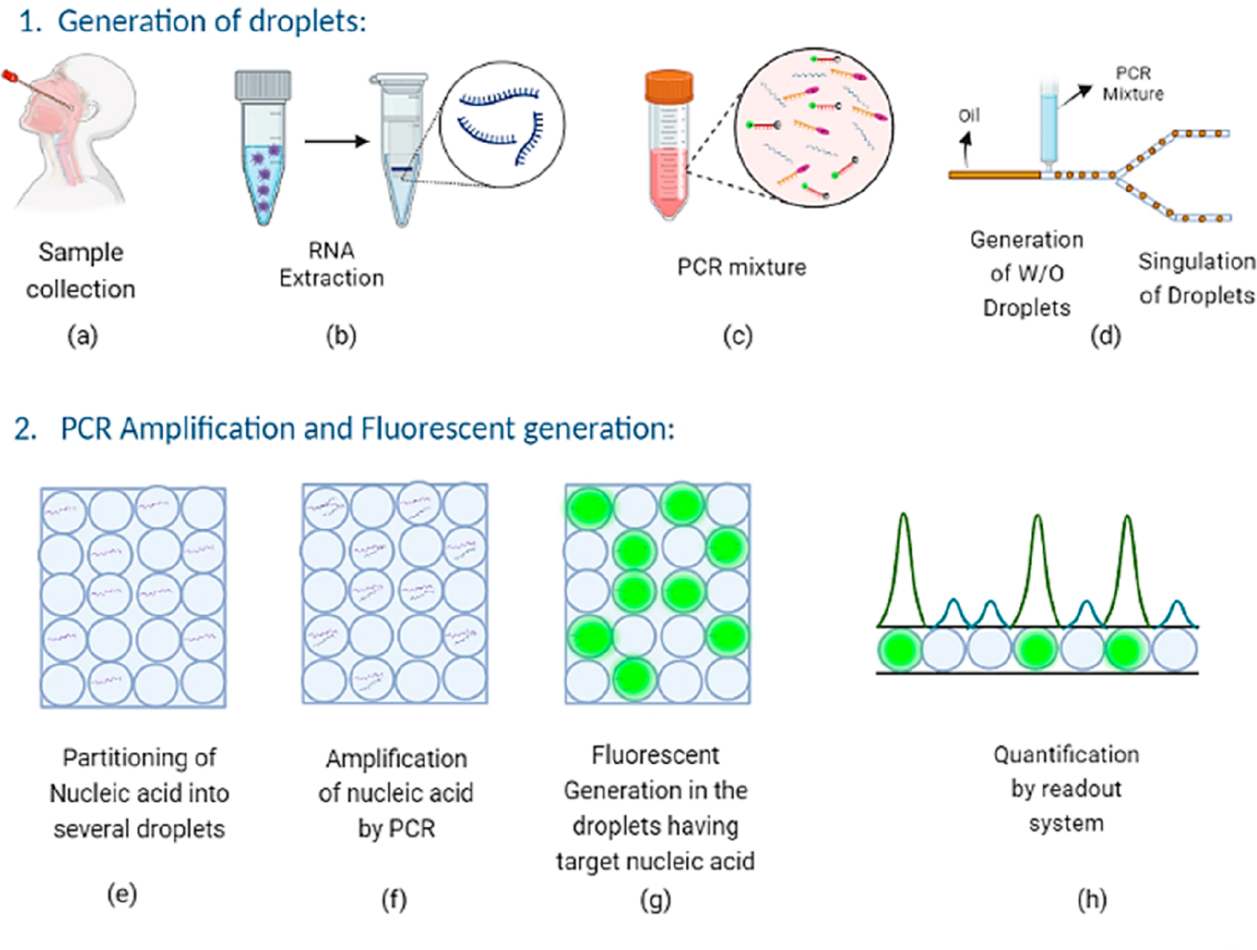
Work flow of Droplet Digital PCR. (a) Sample collection from infected person. (b) Isolation of nucleic acids from sample. (c) Addition of primer, probe, nucleic acid and nucleotides to prepare a PCR mixture. (d) Generation of droplets by mixing PCR mixture with oil. (e) Nucleic acids get distributed in the droplets. (f) Amplification is performed in the droplets (g) During amplification, the polymerase enzyme cleaves the fluorescent labeled probe, which emits fluorescence. (h) Resulting interpretation of droplets by Real-Time Electrochemical detection system. [Created with BioRender.com.]

The use of droplet digital RT-PCR (RT-ddPCR) technique to assess SARS-CoV-2 RNA seems attractive. Like the traditional RT-PCR, this method includes sample collection and nucleic acid extraction. However, in ddPCR, the PCR reaction mixture is broken into 20,000 nanosized droplets before parallel PCR amplification by mixing it with an oil. Each droplet has a distinct collection of target sequences. Instead of doing a single PCR analysis on a single sample, each droplet is treated as a separate PCR sample. Following amplification, the reader isolates all droplets and allows them to flow uniformly for improved detection (Figure 6). A droplet reader is used to measure the fluorescence of each droplet. Positive droplets have more fluorescence, whereas negative drops have background fluorescence. Then, a threshold is set to classify droplets as negative or positive.^56^ Using Poisson statistics, each droplet is considered positive (target existing) or negative (target missing) to determine the absolute target after the reaction. Although target-specific primers and fluorescence-based amplicon recognition are used in real-time and droplet digital RT-PCR, there are some significant distinctions between the two. For instance, real-time RT-ddPCR reagents in reaction chemistry must be appropriate for water-in-oil droplet partitioning and probe chemistry. In contrast, real-time ddPCR commonly employs dark quenchers, and RT-PCR uses fluorescence quenchers. Ideally, guanine should not be present at the 5′ end of ddPCR probes, since it quenches the fluorescence signal even after hydrolysis.^57^ Multiplexing is also possible in ddPCR, as many researchers used different approaches to detect more than one target in a single reaction, such as probe-mixing multiplexing approach in which two different fluorescent probes are used to attach to their specific target and give different fluorescent signal amplitude. The multiplexing method includes two probes with the same fluorescent molecule and gives different fluorescent amplitudes based on the concentration.^58^

The ddPCR technique has been utilized by the Fast Plex Triplex SARS-CoV-2 detection test, in which a microfluidic gadget 1 creates, splits, and collects droplets to amplify the target nucleic acid sequence. Due to the generation of many droplets in the system, most droplets have a single or no target sequence. Irrespective of their concentration in the sample, these sequences can be amplified unbiasedly. Primers and Taqman probes are constructed to target the ORF1ab, N, and RNAase P genes as an internal control.^59^ The DropX-2000 Digital PCR System is used for both droplet formation and droplet PCR thermal cycling. The probes anneal to their exact target sequence. In the extension phase, the Taq polymerase’s 5′ exonuclease activity crushes the probe, allowing the reporter dye to detach from the quencher dye and provide a fluorescence signal. With each cycle, more reporter dye molecules separate from the quencher, intensifying the fluorescence. The probe is a label with reporter FAM targeting the ORF1ab gene, HEX (High Energy X-ray Probe) targeting the N gene, and Cy5 targeting the RNase P gene of SARS CoV-2. Following PCR, the DropX-2000 DScanner (DS4–2000) examines each droplet in three fluorescence channels and the bright area and measures droplet count and diameter for result interpretation. The test has a detection limit of 571.4 copies/mL.^60^

Another gadget based on ddPCR is the Bio-Rad SARS-CoV-2 ddPCR Test, which contains primers and probes that target the nucleocapsid (N) genes N1, N2, and RNase P as internal controls of the COVID-19 virus. The RNA is extracted and transferred to the master mix containing the reverse transcriptase enzyme to convert the RNA into a cDNA strand. The sample and master mix RT-ddPCR mixture is subjected to a QX200/QXDx Automated Droplet Generator to form water in an oil emulsion containing up to 20 000 nL of fractionated droplets. Then, the emulsion is subjected to thermocycling using Bio-RadC1000. After the complete thermocycling, the 96-well RT-ddPCR-ready plate is placed into the QX200/QXDx Droplet Reader. A two-color fluorescence detector is employed after the droplet reader separates the droplets. Each target found using the SARS-CoV-2 genes N1, N2, and the detector scans RP to determine whether droplets are positive or negative. The QuantaSoft Analytical Pro 1.0, QuantaSoft 1.7, and QX Manager 1.1 analysis programs are used in the ddPCR system. The assay’s detection limit was committed to be 150 copies/mL. Primers and probes for recognizing the Nucleocapsid (N) gene N1, N2, and RNase P as internal references are included in the Gnomegen COVID-19 RT-Digital PCR Detection Kit.^61^

### Isothermal Techniques

Isothermal nucleic acid amplification methods allow target nucleic acid sequence amplification at constant temperatures. These methods avoid the high-temperature thermal cycling during PCR, making them more appropriate for laboratories with limited resources and without access to costly, high-energy PCR equipment. Rapid sample preparation and reagent preparation capabilities and the ability to attach a variety of detectors to isothermal detection techniques increase their usability and convenience.^62^

### Loop-Mediated Isothermal Amplification (LAMP) Technique

The loop-mediated Isothermal Amplification (LAMP) technique practices DNA polymerase to amplify target DNA with strand displacement activity. The procedure occurs under isothermal conditions, essential for enabling the precise binding of a primer designed for the target sequence. For target DNA amplification, the approach has excellent efficiency, specificity, and speed.^63^ This technique may precisely identify various parts of the target sequence using four, six, or eight primers. Reverse transcription and LAMP are combined in RT-LAMP to amplify nucleic acids. The RT-LAMP technology is utilized by the Palm Germ-Radar (PaGeR) device. The system contains different heating components for virus deactivation and RT-LAMP reaction. On the PaGeR platform, several tasks could also be accomplished, including the inactivation of the virus, RT-LAMP assay, and result interpretation. The four primers—namely, Forward Inner Prime (FIP), Backward Inner Primer (BIP), Forward Outer Primer (F3), and Backward Outer Primer (B3)—were employed in this RT-LAMP technique to amplify six distinct regions of the target gene.

Although LF and LB, another set of loop primers, could hasten the reaction further, the reaction was performed at 65 °C for 60 min, followed by 2 min of enzyme inactivation at 80 °C. The first step of the reaction is “Strand Invasion”, and the F2 region of FIP binds to its complementary site, F2c, on the strand and initiates the synthesis of the second strand by adding nucleotides complementary to the first strand by the specific polymerase. Then, the F3 binds to the F3c region to dissociate the two strands. Now, the BIP will get attached to the dissociated strand. The same steps will be repeated for BIP. After the reaction of both FIP and BIP, the first product is obtained: a single-stranded RNA with reverse complementary sequences. F1 binds with F1c, forming a loop structure; the same happens with B1, which binds with B1c, and the product gets a dumbbell-shaped structure. All primers can bind to generate more products. After the amplification, various read-out methods are available in Palm Germ-Radar (PaGeR) devices, such as the following:

(1) Readout in real-time: A fluorescent nucleic acid dye, such as Eva Green, could be added to record amplification using real-time quantitative analysis.
(2) Colorimetric measurement: Colorimetric pH indicators such as Malachite Green (MG) are employed in colorimetric measurement for end-point evaluation of LAMP products. Protons (H^+^) are released during amplification due to dNTP binding to the target. The pH is decreased during the LAMP test, which results in a color change from colorless to blue that is discernible to the human eye.^64^
(3) Fluorometric measurement: A fluorescence metal indicator, calcein, is used in place of the colorimetric regent, and Mn^2+^ cations should be added to the reaction mixture; the end-point may be detected using a UV light pen (365 nm).
(4) Dipstick readout on the side: A nucleic acid biosensor could be linked with a lateral flow to display the process results. If the 5′ ends of the LB primer are tagged with biotin and the 5′ ends of the LF primer are tagged with 6-FAM, the LAMP result could create a double-labeled visible product with biotin and 6-FAM (6-6-carboxyfluorescein). The anti-6-FAM antibody on the test line may catch a product with a 6-FAM tag when it runs to the detection region. In contrast, additional antibiotin-Au-NP couples will unceasingly run and be caught by the secondary antibody on the control line.^65^

**Figure 7:**
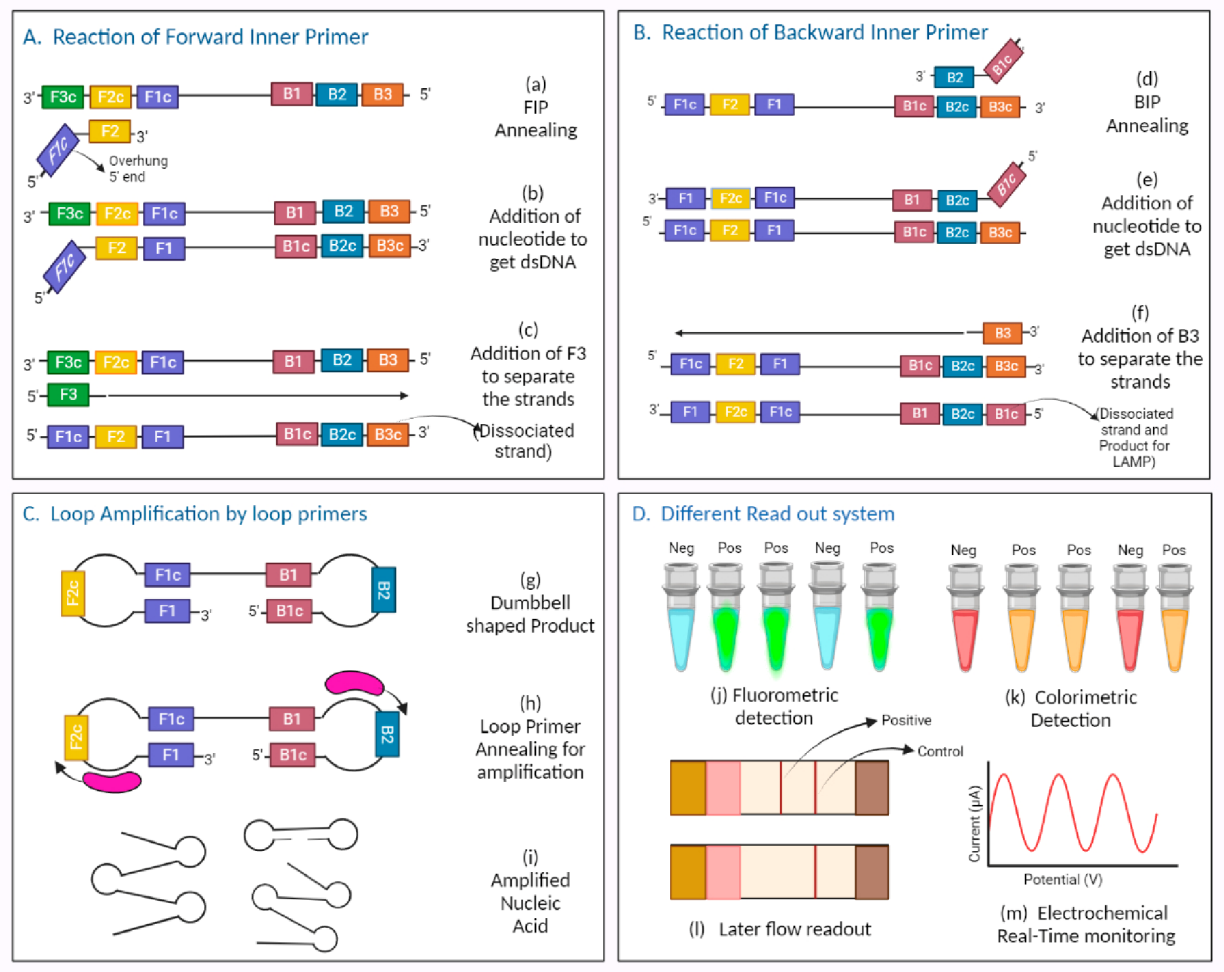
Schematic representation of RT-LAMP technique with its read-out system. (a) Forward Inner Primer (FIP) anneals to denatured ssDNA. (b) FIP initiates the binding of complementary nucleotides to ssDNA. (c) F3 binds to F3c to detach the complementary strand. (d) Backward Inner Primer (BIP) anneals to dissociated strand. (e) BIP initiates the binding of complementary nucleotides to dissociated strand. (f) B3 bind to B3c to detach the complementary strand. (g) F1 binds to F1c and B1 binds B1c convert the strand into dumbbell shape. (h) Loop primers initiates the amplification. (i) Amplified nucleic acid which goes for the detection. (j) Fluorometric detection. (k) Colorimetric detection. (l) Lateral flow read-out. (m) Real-time electrochemical monitoring. [Created with BioRender.com.]

A schematic representation of the RT-LAMP technique with its read-out system is given in Figure 7.

### RT-LAMP with Fluorescence Detection

A fluorescent dye such as SYBR Green or EvaGreen is included in the reaction mixture to enable fluorescence detection in the RT-LAMP technique. As the double-stranded DNA product is synthesized during amplification, these colors intercalate into it. As the amplification process progresses, the dye attaches to the freshly synthesized DNA, causing the fluorescence intensity to rise. A specialized detection device, such as a real-time PCR machine or a fluorometer, is used to measure the fluorescence intensity in real time. During amplification, the equipment regularly monitors the fluorescence intensity, forming a fluorescence curve. The curve may be used to identify whether or not the target RNA sequence was present in the original sample. Alternatively, the amplification can continue for a set amount of time, often 30–60 min, before the reaction is interrupted. At the end point, the fluorescence intensity may be evaluated to detect the presence or absence of the target RNA sequence. This procedure is also known as end-point analysis.^66^

The product based on the principle of RT-LAMP with fluorescent detection is the AQ-TOP COVID-19 Rapid Detection Kit, which employs dual-labeled Peptide Nucleic Acid (PNA) probes. It is in FAM-labeled fluorescence channels that detect the ORF1ab gene of SARS-CoV-2, and HEX fluorescence channels detect human RNAase P, which is utilized as an internal control. Reverse transcriptase and Bst polymerase enzymes are combined in reverse transcription and the LAMP method, which takes place at 60 °C. Fluorescence resonance energy transfer (FRET) probes are inserted into the amplicons to generate the fluorescence. The fluorescence detector on the CFX 96 and ABI 7500 real-time PCR devices may be used to view fluorescence in real time following inclusion. The test also contains positive controls (PC), such as ORF1ab and the human RNase P gene, and negative controls (NC), such as RNase/DNase-free distilled water. The detection limit for the test is 7 copies/μL.^67^ The advanced AQ-TOP COVID-19 Rapid Detection Kit Plus also uses dual-labeled PNA probes, explicitly aiming at SARS-CoV-2 ORF1ab and N genes. PNA is an artificial DNA with a neutral *N*-(2-aminoethyl)-glycine instead of the negatively charged phosphodiester backbone. PNA probes bind to complementary nucleic acids with significantly higher selectivity than DNA detection probes due to their neutrally charged backbone. The AQ-TOP COVID-19 Rapid Detection Kit PLUS has a limit of detection (1 copy/μL), which is significantly lesser when compared to the AQ-TOP COVID-19 Rapid Detection Kit.^68^

Another kit available is the Pro-AmpRT SARS-CoV-2 Test, which is a closed-tube, monoplex RT-LAMP test that employs a real-time approach to assess fluorescence strength for ORF1ab gene amplification by Genie HT instrument and the time to generate 10 000 relative fluorescence units is noted as result output. Instead of using the Ct value to interpret the test results, the technique employs a reaction time cutoff and the amplicon melt profile. As an alternative to IC, this test uses a fluorescent dsDNA intercalating dye to analyze the melt curve following amplification, rather than sequence-specific detection. Identifying nonspecific amplicons with characteristic thermal profiles is the primary goal of the melt curve study. It was discovered that the LoD of the test was 125 genomic equivalents per sample.^69^

Considering the RT-LAMP principle, Zhang et al. also created a pipet-tip-enabled digital nucleic acid analyzer with improved performance for COVID-19 testing by using conventional laboratory equipment and supplies. It is demonstrated that inserting a glass capillary into standard pipet tips allows for the formation of monodisperse, water-in-oil microdroplets using bench-top centrifugation. The SARS CoV-2 ORF1a/b gene, a typical region for testing COVID-19, may be amplified using this method without needing a thermal cycler. After being amplified, the 450–490 nm fluorescence that stimulated the droplets produced positive results. The approach is compassionate, since it can detect 1–10 copies/L.^70^

Another kit developed is the LANTERN-optimized fluorescent-based test, which can be applied to a swab or saliva sample and takes only 30 min to complete. It uses a new sequence-specific detection strategy and depends on the ability of high-fidelity DNA polymerases to proofread. Install and perfect a unique probe from the LAMP primers (namely LF and LB), which primer design programs like Primer Explorer provide as a specificity check to rule out misleading amplicons. LANTERN is a probe that may be connected with any fluorophore or quencher, has no restrictions on target sites, and enables multiplex detection of targets. This is a quick, sensitive, and precise COVID-19 diagnostic assay that has been created by LANTERN technology. This test includes a human internal control located in the same reaction tube and may be applied immediately to swabs or saliva samples.^71^

The COVID-19 virus may be effectively detected within <35 min, using an RT-LAMP test with particle imaging technology and particle diffusometry (PD) on a portable chip device with combined heating. After RT-LAMP, fluorescent bead samples are photographed using a smartphone, and samples with decreased diffusivity are regarded as positive. By utilizing a portable heating unit, it is possible to identify virus particles as low as 30 per mL in an RT-LAMP reaction on a microfluidic chip. The RNA extraction step can also be skipped when utilizing an unprocessed saliva sample for RT-LAMP. SARS-CoV-2-specific RT-LAMP reactions targeting the N and ORF1ab genes must be lyophilized to prevent cold storage. The test conforms with the WHO target product profiles. It is SARS-CoV-2 specific, does not require cold storage, has a detection limit of <35 × 10^4^ virus particles per mL in saliva, and is digitally accessible. For point-of-care screening, the PD-LAMP technology is quick, easy, and attractive.^72^

Iijima et al. used RT-LAMP technology in combination with a Bioluminescent Assay in Real-Time (BART) to identify SARS-CoV-2 and its L452R spike mutation. Compared to traditional real-time RT-PCR, the novel RT-LAMP-BART assay for detecting severe acute respiratory viruses was highly specific. The test discovers 80 copies of the target gene sequence in a sample within 25 min. BART runs on the principle that enzymatic changes of byproducts (PPi) of nucleic acid amplification into ATP by bioluminescence generated by firefly luciferase must be continuously observed.^73^ The time of a particular kinetic signal (a light output peak) generated by the test, which is used to evaluate the results, depends on the target nucleic acid concentration. The LAMP-BART test employs a simple isothermal heating block and a photodiode-based light detector. LAMP-BART hardware is the simplest solution for real-time amplification analysis, since it does not require sophisticated filter sets, thermocycling electronics, or a light source to irradiate the samples. The readers benefit from substantial cost savings as a result of this ease. In addition, the BART reporter system is as expensive as fluorescent reporter systems, and the consumables used in the process are readily available. However, a more expensive fluorescence/quenching probe is not required. The RT-LAMP-BART test can be used for quick and frequent SARS-CoV-2 testing, but it has a limitation in that it is less sensitive than real-time RT-PCR.^74^

An alternative product based on the RT-LAMP technique with fluorescence is the 1 h sample-to-answer nucleic acid test for SARS-CoV-2 that uses inexpensive and commonly available reagents. Viral inactivation and sample lysis are the first two steps of the procedure; after that, there is isothermal amplification of viral RNA using RT-LAMP. Nasal samples were vortexed for 20 s and then heated for 5 min at 95 °C in a heating mantel. The same procedures were employed to treat the saliva sample; however, the heating period was extended to 6 min. The assay has a LoD of 20–23 copies per reaction, targeting the N, E, and ORF1a genes of SARS CoV-2.^75^

Proofreading enzyme-mediated probe cleavage, or Proofman, was used for sequence-specific detection in the LAMP test. This allowed for real-time and visual detection. LAMP may create the dumbbell form in the initial stages of amplification. The products comprising single-strand loops were produced through the exponential amplification phase after the first phase. To accomplish sequence-specific detection, a thermostable proofreading DNA polymerase (Pfu) was inserted along with the Proofman probe, which was tagged at the 3′ and 5′ ends with a fluorophore and quencher. The 3′–5′ exonuclease activity of the Pfu had to be activated by an intentional mismatch at the Proofman probe’s 3′ end. Pfu would cleave the mismatched nucleotide after binding the Proofman probe to the target sequence in the loop domain. The fluorophore is let loose. Thus, the cleaved probe might also serve as an extended primer to improve the effectiveness of isothermal amplification. In real-time analysis, within 50 min, the assay can visualize end points using a transilluminator, providing convenient reporting from the perspective of point-of-care testing (POCT). Furthermore, a one-pot multiplex assay was utilized in addition to different fluorophores to detect multiple SARS-CoV-2 targets and the inner control simultaneously. Serial primers were used to discover an acceptable set for LAMP based on the SARS-CoV-2 N gene to create a strong and visible test for SARS-CoV-2 diagnosis. The limit of detectable template approaches 10 copies of the N gene per 25 L reaction in 40 min at a constant temperature of 58 °C. The N gene’s 12–213th region, which is very conservative and may be utilized as a test for emerging SARS-CoV-2 mutations, contains the LAMP assay primers.^76^

### RT-LAMP with Colorimetric Detection

Reverse transcription followed by colorimetric LAMP in the color assay is a possible way to detect viruses with the naked eye. It utilizes pH-sensitive dye to change the visible color when the reaction pH decreases.^77^ During amplification in dilute buffer solution at 65 °C, nucleotide inclusion by the DNA polymerase releases some byproducts like pyrophosphate group and a hydrogen ion, leading to the change of pH of buffer solution from alkaline to acidic, which leads to a color change. This color change allows the detection of a particular target. In the first iteration of the assay, the two viral primer sets are directed at the SARS-CoV-2 nucleocapsid gene (N) and envelope gene (E). In contrast, they are directed at the spike gene (S) and ORF1a region in the second iteration. A third primer set that specifically targets the human ribonuclease P (RNase P) transcript is used in both test versions as a positive human control. The reaction is complete within 70 min, with a LoD of 0.75 copies/μL.^78^

Based on the principle of RT-LAMP and colorimetric detection, MobileDetect Bio, Inc. developed the Mobile Detect-Bio BCC19 Test, which does DNA amplification at 65 °C. A DNA polymerase with solid strand displacement and replication activity is used in the test, along with a set of primers. The test primers specifically target the N and E genes of SARS-CoV-2. Moreover, cDNA is produced from RNA using a reverse transcriptase polymerase, which is subsequently amplified by a DNA polymerase. Together, these two polymerases enable the simultaneous detection of the virus’s DNA and RNA in the same reaction. The byprodcut of DNA amplification reduces the reaction pH, changing the pH-sensitive dye’s color from red to yellow. This color change indicates the presence of the target sequence. The LoD for this assay is 75 copies/L.^79^

Using two LAMP primer-specific software programs, Tighe et al. also produced 8 primer sets for the SARS-CoV-2 nucleocapsid gene with insertion lengths of 262–945 base pairs (bp).^80^ These amplification lengths are longer than those used in existing techniques, which use insert lengths of 130 or even less and demand an extensive incubation period, different primer and temperature approaches, and specific aides to avoid unwanted amplification. This working model investigation used nasopharyngeal tissue from a patient, pure SARS-CoV-2 RNA, and crude lysate comprising deactivated virus to produce positive RT-LAMP reactions for the target amplicons of 262, 687, 693, and 945 bp. They got permissible colorimetric test results for three primers and concluded that an amplicon size of 500–600 bp could give acceptable results.^80^

The one-step multiplex RT-LAMP test is unique in that it only calls for one step, can be finished within 45 min, and can be kept at room temperature while maintaining diagnostic precision with standard RT-qPCR. The one-step multiplexes colorimetric RT-LAMP, which has a similar LoD (0.65 PFU/mL, Ct = 34.12) to RT-qPCR and is 10 times more sensitive to identify viral RNA in clinical specimens, captures the RdRp, M, and ORF1ab genes. No indication of cross-reactivity with other respiratory illnesses was seen. The diagnostic sensitivity and specificity were 98.6% (95% confidence interval: 94.9–99.8%) and 100.0% (95% confidence interval: 97.4–100.0%), respectively.^81^

### RT-LAMP with Lateral Flow Assay

A lateral flow strip is used as the detection platform in RT-LAMP with lateral flow detection. This strip consists of several zones that facilitate the visualization of the amplified DNA. RT-LAMP with lateral flow detection is a rapid and user-friendly method for nucleic acid detection, with results that can be interpreted visually without requiring specialized equipment. It has found applications in point-of-care diagnostics, field testing, and resource-limited settings. The kit based on this technique is a detection test that targets nucleic acids from the ORF1ab region of the SARS-CoV-2 genome. The Detect test also detects nucleic acids from a human gene, serving as a quality control check for sample assemblage, abstraction, reagent integrity, and test implementation. High-temperature isothermal amplification occurs inside a disposable tube inserted into the recyclable Detect Hub. After amplification, the tube is introduced to the reader, where the liquid flows onto the lateral flow strip. The SARS-CoV-2 and control amplicons attach to colored particles on the lateral flow strip’s sample pad before flowing through the membrane and being arrested by immobilized antibodies at various lines on the strip. An accurate negative result shows the Sample Processing Control line. The SARS-CoV-2 and Sample Processing Control lines must be present in a positive test result. The LoD is 800 copies/mL.^82^

The RdRp and N genes of SARS-CoV-2 were identified through multiplex RT-LAMP, and the mRT-LAMP products were assessed by an AuNP-based lateral flow biosensor (LFB). The optimal test amplification conditions are 63 °C for 30 min. The entire procedure, which consists of the reaction setup, viral RNA extraction, RT-LAMP, and product identification, could be finished within 80 min. The LoD of the mRT-LAMP-LFB technology was 20 copies/reaction. The detection of mRT-LAMP-LFB was 100% specific, with no cross-reactions to additional respiratory infections.^83^

Yang et al. also established an effective and potent technique by integrating a programmable ultraspecific Human Chorionic Gonadotropin (hCG)-link with Toehold-Mediated Strand Exchange (TMSE) probes into standard pregnancy test strips (PTSs). The TMSE probe offers high precision through sequence-specific identification, the RT-LAMP reaction delivers ultrasensitivity via 10^9^–10^10^ amplification, and the PTSs provide an instrument-free and plug-in readout that can be detected visually. Adopting PTSs as a generic signal output can considerably lessen labor costs and failure risk, compared to other lateral flow dipstick (LFD) tests that must be prepared per target sequence. The transduction procedure was successfully integrated into a four-channel, rationally constructed microfluidic point-of-care system to increase mobility. The assay aimed to amplify regions of the M and N genes that are typically conserved. Despite several comparable viruses, including a remarkably similar SARS-CoV-2, it provides the lowest detection concentration of 0.5 copies/μL.^84^

### RT-LAMP with Electrochemical Detection

Early recognition of the infection can be achieved by lowering the detection limit. For this, electrochemical methods play a dynamic role. These fabricated miniature devices are helpful as they offer instant and unfailing results. Electrochemically based biosensors require specific parts, including measurement formats, transducer elements, and biorecognition elements.^85^ The straightforward, economical, and low-cost electrochemical sensor identifies the N and ORF1ab genes of the SARS-CoV-2 genome to support epidemiological monitoring. A disposable test strip with screen-printed electrodes that carry out the RT-LAMP reaction is the foundation for the proposed electrochemical sensor. In this method, first, the nucleic acids are extracted from the collected sample, and a very small amount of sample is dropped on the surface of the custom fabricated screen-printed electrodes (SPEs); RT-LAMP reaction takes place at 63 °C. Methylene Blue is employed in electrochemical detection as a redox intercalator probe to produce a diffusion-controlled current monitored by a portable potentiostat to measure the RT-LAMP reaction. The amplification and monitoring require ∼30 min. A diffusion-controlled current encodes the existence and concentration of RT-LAMP output, such as amplicons and dsDNA. The sensor’s performance is evaluated by considering end-point and time-course data on samples. The electrochemical test strip was discovered to have remarkable reproducibility for the recognition and quantification of RT-LAMP amplicons as low as 2.5 × 10^6^ ng/L.^86^

### Clustered Regularly Interspaced Short Palindromic Repeats (CRISPR)/Cas-Based Tests

The CRISPR/Cas system is an efficient genome-editing tool, because it allows for the fast identification of nucleic acids. This system is a form of adaptive immunity that defends bacteria against viruses by memorizing viral genetic sequences. CRISPR is a region of bacterial DNA that comprises repeated DNA sequences; when a virus invades the bacterium cell, it integrates a small fragment of viral DNA into its own CRISPR area; the integrated fragment is thus referred to as a spacer. This spacer enables the bacteria to remember the virus. The CRISPR also have some CRISPR-associated proteins commonly known as Cas protein, which play a crucial role in identifying and cleaving foreign DNA. In this process, the CRISPR region is first transcribed into the long RNA known as pre-crRNA, which is then fragmented into short crRNA(CRISPR RNA). Each crRNA belongs to a spacer sequence and serves as a guide for the Cas protein. When the bacterium is infected again by a virus, the crRNA attaches to a Cas protein, forming an active CRISPR-Cas complex. The complex searches the viral DNA for a complementary sequence corresponding to the crRNA guidance. If a match is identified, the Cas protein causes a double-strand break in the viral DNA at the target spot, rendering the viral DNA inactive.^87^ Based on the CRISPR-Associated Protein (Cas protein), there are two chief classes of CRISPR/Cas systems: Class 1 (type I/cas3, type III/cas10, and type IV) and Class 2 (type II/Cas9, type V/Cas12, and type VI/Cas13).^88^ CRISPR-based diagnostic techniques function by recognizing a particular sequence linked to an ailment and cutting it to get a readable signal. Class 2 CRISPR-Cas systems are mainly employed for nucleic acid recognition. Cas9 and Cas12 endonuclease enzymes can cut DNA, whereas Cas13 targets RNA. Cas9 becomes inactive after cleaving the target nucleic acid. Cas12 and Cas13 remain active and cleave both target and nontarget sequences. If a sample’s target sequence is present, these enzymes become active and can begin cleaving immediately. As a result, nuclease-mediated destruction of nontarget sequences labeled with a fluorophore dye can provide fluorescence signals that can be used to detect the targeted nucleic acids. Consequently, these nucleases can be exploited for nucleic acid detection.^89^

### CRISPR-Associated Nuclease–13a (Cas13a)

The CRISPR-Cas13a system, utilizing the Sherlock CRISPR SARS-CoV-2 kit, relies on the specific high-sensitivity enzyme reporter unlocking (SHERLOCK) technology. The quencher is a fixed sequence that does not require updating or arranging for different targets, and the CRISPR guide RNA is a brief, inexpensive RNA sequence. Detection media such as a fluorescent plate reader and incubator are less expensive than an RT-PCR apparatus. This Sherlock Biosciences assay became the market’s first FDA-approved CRISPR technology. SHERLOCK technology was developed to sense SARS-CoV-2 N and ORF gene fragments. The LoD values for the N and ORF targets were determined to be 0.9 and 4.5 copies/μL, respectively.^90^

Peng et al. designed a one-pot CRISPR Cas13a-based visual biosensor for speedy and affordable nucleic acid identification. Combining Cas13a cleavage with Recombinase Polymerase Amplification (RPA) in a disposable tube-in-tube vessel prevents contamination in the amplicon. The technique is known as Easy to Use, Contamination-Free, and Stable CRISPR (ECS-CRISPR). First, the RPA reaction is performed in a tube with two aquaphobic holes at the lower surface. The reaction mixture is centrifuged or shaken into an outer tube with the Cas13a reagent packed inside it. The inner and outer tubes are united to form a single reaction pot, allowing nucleic acid testing without removing the cover. Fluorescent visual recognition of target nucleic acid is achieved with blue light-emitting diode (LED) stimulation. The sensor detects virus nucleic acids in 25 min, even without any additional equipment, which fulfils the requirements of nucleic acid detection in resource-limited areas. The results show that the suggested approach has a detection limit of 3 copies/L.^91^

CRISPR/CAS-based colorimetric nucleic acid detection (CASCADE) is a technique for detecting SARS-CoV-2 RNA visually. This technique combines the sensitivity of the CRISPR Cas13a system with ease of colorimetric detection. This can be achieved using specially designed DNA probes that change color upon binding to the target sequence. For example, AuNPs coated with DNA probes can be used. In this kit, after amplification of target nucleic acid by RPA or NASBA, Cas13a first detects and breaks down ssRNA oligonucleotides attached to AuNPs. Their colloidal accumulation thus results, evident in the target sequence’s presence. Without the target sequence, the ssRNA oligonucleotides remain dispersed and the solution appears to be a specific color. However, a visible color change is observed when the target sequence is present. With substantial functionalization of AuNPs, CASCADE can capture picomolar amounts of SARS-CoV-2 RNA. The sensitivity of CASCADE is amplified to the low femtomolar (3 fM) level and maybe even the attomolar (40 aM) level when paired with isothermal nucleic acid amplification (RPA or NASBA), and the AuNP optimization enables a rapid read-out time of 15–30 min.^92^ SARS-CoV-2 and its mutations can also be determined using a CRISPR-Cas13a cascade-based viral RNA assay. Cas13a/crRNA was employed to identify the target RNA of SARS-2, and the detection events drove transcription amplification to make light-up RNA aptamers for fluorescence signal production. Using Cas13a-guide RNA for viral RNA identification guarantees excellent specificity in separating SARS-CoV-2 from SARS-CoV, MERS-CoV, and viral variants. Following CRISPR-Cas13a recognition, a post-transcriptional amplification response was started, resulting in a highly sensitive amplification cascade capable of identifying SARS-CoV-2 RNA with a LoD of 0.216 fM. Additionally, as indicated by N501Y in the SARS-CoV-2 variation, the Cas13C test may discriminate single-nucleotide alterations. This approach was validated by 100% agreement with RT-qPCR data from 12 clinical throat swab specimens. The Cas13C assay can become the standard nucleic acid test for the SARS-CoV-2 virus in resource-limited areas.^93^

### CRISPR-Associated Nuclease–12a (Cas12a)

Mammoth Biosciences and GSK designed DETECTR (DNA Endonuclease-Targeted CRISPR Trans Reporter) to target the SARS-CoV-2 N and E genes with great sensitivity, utilizing the CRISPR-Cas12 technology and visible lateral flow strip. The test lasts 30–40 min and has a LoD of 70–300 copies/mL.^94^

The All-In-One Dual CRISPR-Cas12a assay was also designed for one-pot, ultrasensitive, and visual SARS-CoV-2 identification, which employs a pair of Cas12a-crRNA complexes made by two separate crRNAs to target two different locations of the viral N gene. RPA amplification commences when the AIOD-CRISPR reaction system is incubated in one pot at 37 °C, exposing the Cas12a-crRNA complexes’ binding sites, because of strand displacement. The Cas12a endonuclease becomes active when Cas12a-crRNA complexes combine at the target locations and cleave ssDNA-FQ reporters, causing strong fluorescence signals. The AIOD-CRISPR tool provides a LoD of 4.6–11 copies/μL.^95^

For further simplification of the CRISPR technique, Lee et al. provided the idea of CODA (CRISPR Optical Detection of Anisotropy), which is a process that merged isothermal nucleic acid amplification, CRISPR/Cas12a activation and signal generation in a single assay performed at 42 °C, abolishing the requirement for additional manual processes. Notably, signal identification is based on fluorescence anisotropy measurement, allowing CODA to achieve a better signal-to-noise ratio. Using an integrated heater, optoelectronics, and a microprocessor for data processing, the researcher established a practical, standalone CODA device for point-of-care usage. This approach detects the viral RNA within 20 min after sample application, and the detection limit was 3 copies/μL.^96^

Another advanced tool is the CRISPR/Cas12a-RPA-based one-pot detection approach, which was developed and improved in the presence of target DNA; the procedure involves the reverse transcription of the SARS-CoV-2 RNA to create cDNA, then recombinase polymerase amplifies the resultant DNA and commences collateral activity on a reporter probe using CRISPR/Cas12. Cas12a properties are essential because target DNA sequences and the RNA guide Cas12a could attach to the 20 bp ssDNA target sequence and enhance activation without the utilization of Protospacer Adjacent Motif (PAM) for nonspecific single-stranded DNase (ssDNase) activity. However, PAM is required for dsDNA cleavage. During the RPA process, strand displacement and/or polymerization of ssDNA act as activators for Cas12a. Following that, active Cas12a in the reaction mix cuts ssDNA-FQ reporters, resulting in fluorescence signals to detect the existence of target nucleic acid. The design of the study and guided reaction optimization improve test sensitivity, with only 2 copies of the COVID-19 RNA and 0.5 copies of the COVID-19 DNA detected per microliter.^97^

The UDG-RT-LAMP system (Uracil-DNA-Glycosylase-Reverse 68Transcription-LAMP) system, which is a new method that can successfully remove dUTP-incorporated LAMP amplicons from the CRISPR-LAMP technology, has been established as a way of reducing aerosol adulteration in the CRISPR-LAMP technology. Because of this, UDG-RT-LAMP can altogether remove all traces of earlier amplification from the current amplification process. Cas12a cleavage in LAMP amplicons demonstrated that there were no position restrictions for Cas12a/crRNA identification and cleavage and that the looping location of LAMP amplicons could be easily recognized and cleaved. It can detect FLUA, FLUB, RSVA, RSVB, and spike N501Y or SARS-CoV-2 wild-type in samples containing little more than 10 copies/μL. Infections may be properly diagnosed with this detection method, because of its improved stability and sensitivity in detecting small point mutations.^98^

A digital CRISPR assay for the quantitative detection of SARS-CoV-2 called the Digital Warm Start test (dWS-CRISPR test) was also developed by Ding et al. As the test is initiated at 50 °C and avoids early target amplification at room temperature, dWS-CRISPR makes precise and consistent digital quantification possible. By focusing on the SARS-CoV-2 nucleoprotein gene, the dWS-CRISPR assay can identify SARS-CoV-2 RNA in the chip at a meager quantity of 5 copies/L. It does not require RNA extraction and can identify coronaviruses in hot saliva samples. As a result, the DWS-CRISPR approach, which is a trustworthy and sensitive CRISPR test, enables precise SARS-CoV-2 identification for digital quantification.^99^

To increase the affordability of the CRISPR/Cas12a instrument, Han et al. developed a CRISPR/Cas12a-based electrochemical apta sensor. This provided the quick, inexpensive, and sensitive SARS CoV-2 nucleocapsid protein (Np) quantification. An electrochemical detecting interface was made by immobilizing a Methylene Blue-tagged polyadenine DNA sequence (polyA-MB electrochemical reporter) on a gold electrode surface. Second, an Np aptamer and an activator strand were combined to create an arched probe, and, in the presence of COVID-19 Np, a specific interaction between aptamer and target allowed the activator strand to be free from the arched probe, activating the CRISPR system’s trans-cleavage activity. Afterward, the polyA-MB reporters were removed from the electrode surface, reducing the differential pulse voltammetry (DPV) current at a potential of −0.27 V (against Ag/AgCl). It is practicable to sense SARS CoV-2 Np at LoD = 16.5 pg mL^–1^, using an electrochemical apta sensor developed using CRISPR/Cas12a technology. The test may be finished within <30 min. The apta sensor is also very selective for other proteins, as shown by the studies.^100^

RApid VIsual CRISPR (RAVI-CRISPR) is also a field deployable isothermal amplification system that uses a ROX-labeled reporter with CRISPR/Cas12a to identify five crRNAs for the E gene (E-crRNA-1 to E-crRNA-5) and nine crRNAs for the N gene (N-crRNA-1 to N-crRNA-9). RAVI-CRISPR is an instrumentless, single-tube colorimetric POCT. For incubation, it only needs a transportable rechargeable hand warmer. SARS Coronavirus 2 and the African Swine Fever virus were successfully detected with great sensitivity using RAVI-CRISPR. The LoD is a total of 40 copies.^101^

Another affordable, easy-to-interpret tool is CRISPR-top, which combines synchronous target preamplification with coupled CRISPR/cas12b-mediated identification in a single-pot reaction medium at a persistent temperature. CRISPR-top targets the SARS-CoV-2 ORF1ab and NP (nucleoprotein) genes and runs for 40 min at 59 °C with little equipment. The COVID-19 CRISPR-top test can produce results within only 60 min and is easy to interpret, utilizing fluorescence or lateral flow readouts. COVID-19 CRISPR-top has an analytical LoD of 10 copies per reaction (for each detection target), with no cross-reactivity identified with non-SARS-CoV-2 templates. The assay’s specificity was 100% among clinically derived non-COVID-19 samples.^102^

### CRISPR-Associated Nuclease-9 (Cas9)

The FnCas9 Editor Linked Uniform Detection Assay (FELUDA) practices a direct Cas9-based enzymatic detection to recognize nucleotide sequences without requiring a trans-cleavage of the reporter molecule. The researchers presented the JATAYU web tool to assist end users and revealed that FELUDA is 100% precise in analyzing single nucleotide variations (SNVs), particularly heterozygous carriers. FELUDA is a semiquantitative tool that can be utilized for various purposes, including molecular diagnostics in infectious disease occurrences like COVID-19. It is also adaptable to a wide range of signal-detecting systems. FELUDA, which employs a lateral flow readout, offers 100% sensitivity and 97% specificity for all viral load levels in 1 h. Using RT-RPA and the mobile app True Outcome Predicted through Strip Evaluation (TOPSE), they demonstrate a prototype of FELUDA for CoV-2 recognition closer to home.^103^

The alternative reliable pathogen detection method Bio-SCAN, known as a biotin-coupled specific CRISPR-based assay for nucleic acid identification, does not require specialized equipment or technical expertise. From sample collection to conclusion, it requires only 60 min. Bio-SCAN can identify the SARS-CoV-2 genome. The target nucleic acid sequence is precisely recognized on lateral flow strips in the first phase using biotin-labeled SpCas9 after being isothermally amplified in 15 min, utilizing recombinase polymerase amplification (dCas9). The end outcome can be seen with the unaided eye. In contrast to currently used CRISPR-Cas-based pathogen recognition methods, Bio-SCAN does not require supplementary reporters, enhancers, probes, reagents, or classy instruments to understand the data. Bio-SCAN is extremely sensitive and ultimately distinguishes 4 copies/L of synthetic SARS-CoV-2RNA genome, which is a therapeutically relevant level.^104^

### CRISPR-Associated Nuclease-3 (Cas3)

Cas3-Operated Nucleic Acid Detection (CONAN) is a quick, inexpensive, and instrument-free detection approach that detects SARS-CoV-2 in clinical specimens and single-base-pair mutations in IAV variants. The SARS-CoV-2 N (nucleoprotein) gene regions (N1 and N2) are amplified.^105^

### Rolling Circle Amplification (RCA)-with MALDI-TOF-MS

Rolling circle amplification (RCA) is a molecular biology method for amplifying circular DNA molecules. It is excellent for multiplying tiny circular DNA templates like plasmids, circular virus genomes, or circularized DNA fragments. It is an isothermal enzymatic method that employs a circular DNA template and specific DNA or RNA polymerases to construct a long ssDNA or RNA from a short DNA or RNA primer. RCA relies on the activity of a DNA polymerase enzyme with strand displacement capability. The activity of a DNA polymerase enzyme with strand displacement capacity is required for RCA. The process begins when the short DNA primer binds to the circular DNA template at a particular place. Then, the DNA polymerase enzyme begins the unidirectional addition of complementary nucleotides along the circular DNA. The addition of a complementary nucleotide causes the original strand to be displaced, and DNA polymerase continues synthesizing the new DNA strand complementary to the circular template. Consequently, a lengthy, single-stranded DNA molecule with numerous initial circular template sequence repeats is formed. After getting the dsDNA, it proceeds through the RCA cycle again, resulting in significant amplification. RCA can amplify tiny amounts of circular DNA, making it suitable for samples with limited starting material. This technology has been utilized to provide sensitive diagnostic procedures for various targets, including DNA and RNA, proteins, and cells.^106^

Han et al. provide a three-way junction-induced exponential rolling circle amplification (3WJ-eRCA) integrated MALDI-TOF MS approach for SARS-CoV-2 RNA targeting. The method is advantageous in terms of speed, specificity, and efficiency, and it can sense many genes for SARS-CoV-2 identification at the same time. SARS-CoV-2 was distinguished with remarkable specificity from other coronaviruses, including SARS-CoV, bat SARS-like coronavirus (bat-SL-CoVZC45), and MERS-CoV. Using an isothermal technique (55 °C), the method will target a specific nucleocapsid (N) and the virus’s ORF1ab genes in a single test within <30 min. The construction of such three-way junctions is an essential step in fabricating MALDI-TOF MS amplification products. The 3WJ template is constructed with a rolling circle primer sequence at the 5′ ends and a complementary sequence to the COVID-19 viral target sequence at the 3′ ends, separated by the active site of the Nt.BspQI nicking endonuclease and a complementary sequence of 3WJ-primer components. A 7-base sequence in the 3WJ-primer can be linked to the 3WJ template. The 5′ ends of the 3WJ-primer targets the SARS-CoV-2 RNA. Due to a lack of complementarity between the 3WJ-primer and the 3WJ-template, the junction structure does not become stable without the SARS-CoV-2 target RNA. If the SARS-CoV-2 target RNA is extant, the 3WJ template and 3WJ-primer hybridize stable formation of the three-way junction structure. Many trigger strands of RC primers are produced due to several rounds of elongation and cleavage of a 3WJ-primer using DNA polymerase Vent (exo-) and nicking endonuclease Nt.BspQI.^107^

For further advancement, in 2023, Li et al. designed an ingenious dual-gene-controlled rolling circle amplification technique for further advancement. This technique can detect N and RdRp genes simultaneously and provide results with an electrochemical detection method. The simultaneous detection of the two genes can be appreciated, which guarantees the accuracy and specificity of the analysis of SARS-CoV-2 viruses.^108^

### Nicking Enzyme Amplification Reaction (NEAR)

NEAR is an isothermal amplification method driven by the collaborative activity of a nicking enzyme and DNA polymerase strand-displacing action. Nicking enzyme, when added to the reaction, creates a small nick at the specific site of the target DNA. The nicked DNA strand serves as the starting point for DNA synthesis. A DNA polymerase with strand displacement activity is added to the reaction mixture, extending the nicked strand, displacing the other strand. Thousands of amplicons can be created from a single primer due to repetitive nicking, and polymerization with strand displacement may occur at a single restriction site, giving a high amplification productivity.^109^

Abbott Diagnostics (Chicago, IL, USA) has developed the ID NOW COVID-19 assay, which is an isothermal NAAT that uniquely targets the distinctive region of the RdRp gene. The device is perfect for a “near-patient” test in emergency rooms, since it uses dry nasal swabs, is simple to operate, and does not require complicated equipment. A positive result can be completed within 5 min, whereas a negative result can take up to 13 min.^110^ An ID NOW instrument, a sample receiver with elution/lysis buffer, two closed reaction tubes carrying a lyophilized pellet in each, a test base, and a transfer cartridge for moving the eluted sample to the test base are all included. The reaction tubes of the test base contain SARS-CoV-2 amplification reagents and internal control. The primer-like templates that target SARS-CoV-2 RNA amplify a particular RdRp segment region. Molecular beacons with fluorescent labels are used to find each of the amplified target RNAs individually. The sample receiver and test base are added to the ID NOW Instrument to carry out the test. Target amplification starts when the sample is put in the sample receiver and sent via the transfer cartridge to the test base. The gadget delivers heating, mixing, and detection. The test can identify up to 125 genomic equivalents per milliliter.^111^

### Transcription-Mediated Amplification (TMA)

TMA entails reverse transcription-based isothermal rRNA amplification and RNA polymerase-based multiple transcript synthesis. These RNA copies are then amplified, hybridized with a complementary oligonucleotide probe, and labeled with a chemiluminescent tag to enable detection. TMA typically creates 100–1000 copies per cycle and causes an increase by a factor of 10 billion within 15–30 min.^112^ Hologic’s Aptima SARS-CoV-2 Assay employs TMA, while direct RNA amplification requires reverse transcriptase with the T7 RNA polymerase enzyme. Several authorized NATs based on TMA use the Aptima SARS-CoV-2 Assay, which is the Poplar SARS-CoV-2 TMA Pooling Assay,^113^ the Quest Diagnostics HA SARS-CoV-2 Assay,^114^ the Let us Get Checked Coronavirus Assay (COVID-19) TEST,^115^ and the Procleix SARS-CoV-2 Assay,^116^ which detects ORF1ab genes in two regions and an IC, which is intended to be carried out using the Panther System. The target RNA molecule is extracted with the help of a capture oligomer with a sequence complementary to the target protein’s specific region and a chain of deoxyadenosine residues. During hybridization, the sequence-specific portions of the capture oligomers bind to particular portions of the target molecules. The reaction temperature is maintained at room temperature to remove the captured oligomer-target complex from the solution. These temperature dips allow hybridization between the polydeoxythymidine molecules covalently bound to the magnetic particles and the deoxyadenosine region of the capture oligomer. The magnetized microparticles stuck to the side of the reaction vessel with the target molecules are attached, and the supernatant is aspirated. Any leftover specimen matrix that can contain amplification reaction inhibitors is removed by cleaning the particles. After the target capture stage is complete, the samples are ready for amplification. TMA carries out the amplification, and the RNA amplicons are identified by hybridization with chemiluminescent nucleic acid probes labeled with various acridinium ester molecules. As part of the detection process, light emitted from the tagged hybrids is detected in the luminometer as photon signals and measured in relative light units (RLU). The results are interpreted using the total photon output (RLU) and the kinetic curve type. LoD for the Aptima SARS-CoV-2 Assay is 83 copies/mL^117^ (Table 5). The Aptima SARS-CoV-2/Flu assay works on the same principle as Aptima SARS-CoV-2 Assay. The same reaction detects Influenza A, Influenza B, and SARS-CoV-2. SARS CoV-2 has two ORF1ab gene segments. The SARS CoV-2 gene is recognized in the Panther system, using the FAM fluorescent channel. Influenza A is detected using ROX, and Influenza B is detected using the HEX fluorescent probe.^118,119^

**Table 5:** Target Gene and Limit of Detection (LoD) of Various Nucleic Acid Detection Tests

name of the test	target gene	limit of detection, LoD	ref
TaqPath COVID-19 Fast PCR Combo Kit 2.0	ORF1a, ORF1b, N gene	1000 GCE/mL	(123)
**Real-Time RT-PCR**			
CDC 2019-nCoV Real-Time RT-PCR Diagnostic Panel (CDC)	N1, N2	10^0^ RNA copies/μL (Qiagen DSP); 10^0.5^ RNA copies/μL (Qiagen EZ1)	(36)
**Multiplex RT-PCR**			
The SARS-CoV-2 (Flu SC2) Multiplex Assay	N	1.01 × 10^–4^^2^ TCID50/mL (TaqPath 1-step Multiplex); 5.06 × 10^–2^ TCID50/mL (ΜLtraplex 1-step ToughMix)	(120)
Roche’s cobas SARS-CoV-2 & Influenza A/B	ORF1a/b, E	0.12 TCID50/mL	(41)
Xpert Xpress SARS-CoV-2/Flu/RSV	E, N2	131 copies/mL	(40)
QIAstat-Dx Respiratory SARS-CoV-2 Panel	ORF1b, E	500 copies/mL	(121)
Alinity m Resp-4-Plex Assay	RdRp, N	0.005 TCID50/mL (30 GE/mL)b	(122)
**RT-PCR with Lateral Flow**			
AccμLa SARS-CoV-2 Test	N	200 copies/reaction	(46)
**RT-PCR with Electrochemical Detection**			
ePlex Respiratory Pathogen Panel 2	NR	250 GC/mL	(48)
ePlexSARS-CoV-2 Test	NR	750 GC/mL	(51)
**RT-PCR with MALDI-TOF Detection**			
MassARRAY	ORF1ab, N1, N2, N3	2.5 copies/μL	(54)
SARS-CoV-2 Panel	ORF1		
SARS-CoV-2	ORF1ab, N1, N2, N3	0.69–2.75 copies/μL	(124)
MassArray Test	ORF1		
Ethos Laboratories	ORF1ab, N1, N2, N3	1 TCID50/mL	(125)
SARS-CoV-2 MALDI-TOF Assay	ORF1		

**qSTAR**			
LumiraDx SARS-CoV-2 RNA STAR Complete	ORF1a	7500 copies/mL	(55)
LumiraDx SARS-CoV-2 RNA STAR	ORF1a	500 copies/mL	
**RT-Digital PCR**			
FastPlex Triplex SARS-CoV-2 detection kit (RT-Digital PCR)	ORF1ab, N	571.4 copies/mL	(60)
Bio-Rad SARS-CoV-2 ddPCR Test	N1, N2	150 copies/mL	(126)
Gnomegen COVID-19 RT-Digital PCR Detection Kit	N1, N2	8 GC/reaction	(61)
**Loop-Mediated Isothermal Amplification (LAMP) Technique**			
PaGeR devise with RT-LAMP.	ORF1ab, N	1 copy/μL	(65)
**RT-LAMP with Fluorescence Detection**			
AQ-TOP COVID-19 Rapid Detection Kit PLUS	ORF1ab, N	1 copy/μL	(68)
AQ-TOP COVID-19 Rapid Detection Kit	ORF1ab	7 copies/μL	(127)
Pro-AmpRT SARS-CoV-2 Test	ORF1ab	125 GE/swab	(69)
UOL COVID-19 Test Kit	NR	400 copies per swab	
digital LAMP or dLAMP	ORF1ab	1–10 copies/μL	(128)
LANTERN		30 virus particles per mL	(71)
RT-LAMP with particle diffusometry (PD)	ORF1ab, N	35 × 10^4^ viral particles per mL	(72)
RT-LAMP-BART	*RdRp*, L452R spike mutation	80 copies/reaction	(74)
Sample-to-answer, extraction-free, real-time RT-LAMP test	N, E, ORF1a	20–23 copies/reaction	(75)
**RT-LAMP with Colorimetric Detection**			
MobileDetect (MD-Bio BCC19) Test Kit	E, N	75 copies/μL	(79)
Color Genomics SARS-CoV-2 RT-LAMP Diagnostic Assay	ORF1ab, E, N	0.75 copies/μL	(129)
RT-Proofman-LAMP	ORF1ab, N	100 copies/reaction	(130)
one-step multiplex and colorimetric RT-LAMP using lyophilized LAMP reagent	ORF1ab, RdRp, M	0.65 PFU/mL	(81)
**RT-LAMP with Lateral Flow Assay**			
Detect Covid-19 Test	ORF1ab	800 copies per ML	(82)
(mRT-LAMP-LFB) Multiplex reverse transcription loop-mediated isothermal amplification linked to a nanoparticle-based lateral flow biosensor	RdRp, N genes	20 copies per reaction	(83)
RT-LAMP with pregnancy test strips PTSs	M or N genes	0.5 copies/μL	(131)
**RT-LAMP with Electrochemical Detection**			
RT-LAMP with screen-printed electrodes	ORF1ab, N	2.5 × 10^6^ ng/L	(86)
**RT-LAMP with CRISPR Base Test: Cas13a**			
Sherlock CRISPR	ORF1ab, N	4.5 copies/μL	
SARS-CoV-2 Kit			
ECS-CRISPR		3 copies/μL	
CASCADE (CRISPR/CAS-Based Colorimetric Nucleic Acid DEtection)	ORF1ab	10^6^–10^4^ viral copies/mL	(92)
**RT-LAMP with CRISPR Base Test: Cas12a**			
SARS-CoV-2 DETECTR Reagent Kit	N, E	70–300 copies/mL	(94)
All-In-One Dual CRISPR-Cas12a (AIOD-CRISPR) Assay for One-Pot	N	4.6–11 copies/μL	(95)
CODA (CRISPR Optical Detection of Anisotropy)	N1, N2	3 copies/μL	(132)
Statistical Design of Experiments (DoE) CRISPR/Cas12a-RPA Assay	N	2 copies/μL for COVID-19 RNA	(97)
		0.5 copies/μL for COVID-19 DNA	
CRISPR-LAMP Technology (a Uracil-DNA-Glycosylase-Reverse Transcription-LAMP System)	spike N501Y	10 copies/μL	(98)
Digital Warm-Start CRISPR (dWS-CRISPR)	N	5 copies/μL	(99)
CRISPR/Cas12a-Electrochemical Aptasensor	N	16.5 pg mL^–1^	
RApid VIsual CRISPR (RAVI-CRISPR)	N	2 copies/μL of full-length COVID-19 genome and 0.5 copy/μL of DNA fragment of N gene was visually detected	(101)
**RT-LAMP with CRISPR Base Test: Cas12b**			
CRISPR-top (**CRISPR**-Mediated **t**esting in **o**ne-**p**ot	ORF1ab, N	10 copies/reaction	(102)
**RT-LAMP with CRISPR Base Test: Cas9**			
FELUDA (FnCas9 Editor Linked Uniform Detection Assay)	S, N	NR	(103)
Bio-SCAN (Biotin-Coupled Specific CRISPR-Based Assay for Nucleic Acid Detection)	ORF1a, ORF1b, S, Spike. E, envelope. M, membrane. N, nucleocapsid genes	4 copies/μL	(133)
**RT-LAMP with CRISPR Base Test: Cas3**			
(CONAN) Cas3-Operated Nucleic Acid Detection	N1, N2	>1.0 × 10^10^ copies for the activator	(105)
**Rolling Circle Amplification (RCA)-with MALDI-TOF-MS**			
3WJ-eRCA (Three-Way Junction-Induced Exponential Rolling Circle Amplification)	ORF1ab, N	NR	
**Nicking Enzyme Amplification Reaction (NEAR)**			
ID NOW COVID-19 Assay	RdRp	125 GE/mL	(111)
**Transcription-Mediated Amplification (TMA)**			
Aptima SARS-CoV-2 Assay	2 regions of ORF1ab	83 copies/mL	(117)
Poplar SARS-CoV-2 TMA Pooling Assay	2 regions of ORF1ab	83 copies/mL	(113)
Quest Diagnostics HA SARS-CoV-2 Assay	2 regions of ORF1ab	83 copies/mL	(114)
Lets Get Checked Coronavirus (COVID-19) Test	2 regions of ORF1ab	83 copies/mL	(115)
Procleix SARS-CoV-2 Assay	2 regions of ORF1ab	60 copies/mL	(116)
The Aptima SARS-CoV-2/Flu Assay	2 regions of ORF1ab, 1 region of the Matrix gene for Flu A, and Flu B.	1.8 × 10^2^ NDU/mL	(119)

## Discussion

SARS-CoV-2 is a viral particle that affects the entire world and disrupts the existence of all living creatures. The virus became increasingly lethal as a result of a lack of diagnostic techniques, prevention measures, and treatment alternatives, killing billions of people globally. After considering the severity of the repercussions, WHO declared this novel coronavirus a pandemic. Its fast mutation and asymptomatic instances prompted the development of improved, quick, and precise diagnostic procedures. Various antigen-detecting methods have been developed to aid in the early identification of viruses. This Review focuses on the very precise Nucleic Acid Amplification Test (NAAT), which reads out the sequence unique for SARS-CoV-2 in a specific nucleic acid and amplifies it for rapid and reliable results. In the early phases of the pandemic, RT-PCR has been referred to as the gold standard for detecting SARS-CoV-2. The approach entails converting viral RNA into cDNA and amplifying it for reliable measurement. To interpret the outcomes of amplification, many detection methods were devised. One of the most well-known detection approaches is fluorescence detection, which includes using fluorescently tagged probes to detect the presence of a specific DNA fragment. The fluorescently tagged probe hydrolyses and emits fluorescence as the PCR cycle progresses, indicating the presence of antigenic sequence, and the intensity of the fluorescence may be used to determine the quantity of viral nucleic acid. As the sensitivity of this method is only limited to the SARS-CoV-2 genome, it was urgently required to develop a technique which can also focus on all volatile organic compounds (VOCs) of coronavirus and spike mutations. Therefore, Roberto Monroy-Contreras et al. suggested Multiplex RT-PCR, which can sense all SARS-CoV-2 VOCs and its mutations. It is difficult to design fluorescent-based instruments at low cost; therefore, some portable devices were required, so many new techniques came into existence. An emerging technique called RT-PCR with lateral flow involves the identification of analytes in a test strip. The principle of this method is based on the antigen–antibody reaction. This is the easiest method of detection, which does not include sophisticated instrumentation. The results can be obtained within 30 min. RT-PCR with electrochemical detection is another accurate technology in which all processing, from RNA extraction to identification, is done in a single cartridge. The target DNA is first integrated with a ferrocene-labeled probe. Still, once the capture probe is introduced to the procedure, the ferrocene-labeled probe detaches from the target DNA, resulting in the hybridization of the target DNA and capture probe. The free ferrocene then aids in electron transport to the electrode, generating electrical signals. The creation of these electrical impulses indicates the presence of target protein. A new approach, MALDI-TOF MS technology, can create mass spectra from nasopharyngeal swabs and can identify many mutations in viruses and detect different strains, assisting in the speedy and exact detection of viral infection. However, it is not widely used because its processing time is ∼9 h. Aside from RT-PCR, various additional procedures may deliver results in minutes, such as qSTAR, which combines the extraction and amplification stages into one, reducing processing time. To obtain the results of higher precision and increasing robustness, Droplet Digital PCR has shown outstanding results. This approach divides the reaction mixture into 20 000 nanodroplets to get quantitative findings. ddPCR outperforms classical RT-PCR because it can detect multiple target genes while reducing equipment costs and processing time. However, all of these approaches need thermocycling; hence, to carry out the reaction at room temperature, isothermal nucleic acid amplification tests were used. One of the most used isothermal procedures is reverse transcription-loop mediated isothermal amplification, performed at 65 °C and employing 4–6 primers to achieve more-precise results. It also takes 30–60 min to complete. RT-LAMP, like RT-PCR, may be detected using various readout methods such as fluorescence, dipstick assessment, colorimetric measures, etc. Several devices in this Review employ RT-LAMP with fluorometric detection, such as the AQ-TOP COVID-19 Rapid Detection Kit, which uses a peptide nucleic acid probe for greater specificity and has a detection limit of 7 copies/μL. But the superior AQ-TOP COVID-19 Rapid Detection Kit Plus technique has an LoD of 1 copy/μL. Another technique, called the Pro-AmpRT SARS-CoV-2 Test, utilizes an amplicon melt profile to interpret the results and utilizes a fluorescent dsDNA intercalating dye to analyze the melt curve following amplification. A pipet-tip-enabled digital nucleic acid analyzer uses conventional laboratory supplies and gives excellent fluorescence by forming monodisperse water in oil microdroplets. Multiplex target detection has been enabled by the LANTERN-optimized fluorescent-based test, which is a quick, sensitive, and highly specific technique. Merging the RT-LAMP technology with particle diffusometry reduced the processing time to 35 min. It does not require cold storage, has a detection limit of <35 × 10^4^ virus particles per mL in saliva, and is digitally accessible. The PD-LAMP technology is quick, easy, and attractive for point-of-care screening. SARS-CoV-2 and its L452R spike mutation are identified using RT-LAMP technology, in conjunction with a Bioluminescent Assay in Real-Time (BART). In 25 min, the test detects 80 copies of the target gene sequence in a sample. The RT-LAMP-BART test can be used for rapid and frequent SARS-CoV-2 testing; however, it is less sensitive than real-time RT-PCR. Sequence-specific detection has also been possible by utilizing a proofreading enzyme-mediated probe, enabling visual and real-time analysis. Visible analysis is also feasible using modern approaches such as in RT-LAMP with colorimetric measurement, including a pH indicator dye, to give a color change in the reaction after amplifying the target sequence. Another highly sensitive approach requiring minimum target for detection is RT-LAMP with lateral flow assay, which includes the RT-LAMP amplification product to flow in a lateral flow strip and allows the visual detection. The test also enables multiplex target detection by merging RT-LAMP with Lateral Flow Biosensors. RT-LAMP technique with electrochemical detection is also a cost-effective technique that utilizes disposable test strips with screen-printed electrodes and generates results within only a few minutes. CRISPR, a gene editing technique, is also used in viral recognition. Because these approaches can detect viral RNA at low concentrations, they can diagnose infection in its early stages. The Sherlock CRISPR SARS-CoV-2 kit was the first FDA-approved CRISPR technology on the market. Another one-pot CRISPR Cas13a-based visual biosensor for visual analysis was developed later. CASCADE methods can detect RNA in femtomolar to attomolar amounts. Mammoth Biosciences and GSK designed DETECTR for easy analysis, which uses the CRISPR-Cas12 technology and visible lateral flow strip to identify the target. For reducing processing steps, a modern approach called CODA merged isothermal nucleic acid amplification, CRISPR/Cas12a activation, and signal generation in a single assay performed at 42 °C. This approach detects the viral RNA within 20 min after sample application, and the detection limit was 3 copies/μL. Spike N501Y mutation can also be identified by the advanced technique known as uracil-DNA-glycosylase-RT-LAMP. The dWS-CRISPR assay can identify SARS-CoV-2 RNA in the chip at a meager quantity of 5 copies/L. It does not require RNA extraction and can identify coronaviruses in hot saliva samples. RAVI-CRISPR is also an instrument-less, single-tube colorimetric POCT. The FnCas9 Editor-Linked Uniform Detection test (FELUDA) and Biotin-coupled selective CRISPR-based test (Bio-SCAN) are also described in the study. Using Bio-SCAN, the ultimate result is seen with the naked eye, and it does not require additional reporters, enhancers, probes, chemicals, or fancy devices to comprehend the data. Cas3-Operated Nucleic Acid Detection (CONAN) is described for identifying single-base-pair alterations in IAV variants. Aside from these RT-LAMPs, several alternative isothermal methods have been described. A fantastic approach known as 3WJ-eRCA has brought about a revolution by identifying numerous genes for SARS-CoV-2 identification at the same time and being able to differentiate SARS-CoV-2 from other coronaviruses. The Nicking Enzyme Amplification Reaction (NEAR) is another isothermal approach that employs dry nasal swabs; it is simple to perform and requires no expensive equipment. This method is ideal for testing in emergencies, because a positive result may be obtained within 5 min. Transcription-mediated amplification generally produces 100–1000 copies every cycle, resulting in a 10-billion-fold increase in 15–30 min. This Review has highlighted many nucleic acid amplification tests capable of meeting the gold standard for identifying the SARS-CoV-2 virus, and it has provided an idea for further research into such approaches. Focusing on ways that can produce precise results in the shortest amount of time and at the earliest stage of infection will help to avoid the uncontrolled spread of such deadly viruses.

## Conclusion

The nucleic-acid-based diagnostic methods that are now being developed for the detection of COVID-19 and that may one day be approved for the detection of additional diseases were covered in this Review. Early discovery and isolation of positive cases are crucial to controlling infection outbursts. For this reason, creating quick diagnostic methods is essential to discontinue the transmission of viruses at their first stages. The review elaborated on various techniques, including nucleic acid amplification tests and rapid detection of COVID-19 pathogens. The study systematically elucidated various diagnostic kits available for the detection of viruses and also gave insight into the detection of various other viruses by using nucleic acid amplification tests.

## ABBREVIATIONS

3CLpro: 3C-like Protease
3WJ-eRCA: 3-Way Junction-Induced Exponential Rolling Circle Amplification
ACE2: Angiotensin Converting Enzyme-2
AIOD-CRISPR: All In One Dual-CRISPR Amplification
AuNPs: Gold Nanoparticles
B3: Backward Outer Primer
BART: Bioluminescent Assay in Real Time
Bio-SCAN: Biotin Coupled Specific CRISPR-Based Assay for Nucleic Acid
BIP: backward inner primer
bp: base-pair
Cas: CRISPR-associated nuclease
CASCADE: Cas-Based Colorimetric Nucleic Acid Detection
CDC: Centers for Disease Control
cDNA: complementary DNA
CODA: CRISPR Optical Detection of Anisotropy
CONAN: Cas3 Operated Nucleic Acid Detection
CRISPR: Clustered Regularly Interspaced Short Palindromic Repeats
CRISPR-top: CRISPR-mediated Testing in One Pot
Ct: cycle threshold
CTD: C-Terminal Domain Detection
DETECTR: DNA Endonuclease-Targeted CRISPR Trans Reporter
DoE: Design of Experiment
DPV: differential pulse voltameter
dsDNA: double-stranded DNA
dws-CRISPR: Digital Warm-Start CRISPR
E: Envelop protein
EUA: Emergency Use Authorization
F3: Forward Outer Primer
FAM: fluorescein amidites
FDA: Food and Drug Administration
FELUDA: FnCas9 Editor Linked Uniform Detection Assay
FIP: Forward Inner Primer
FL: fusion loop
FRET: fluorescence resonance energy transfer
hCG: Human Chronic Gonadotropin
HE: Haemagglutinin-esterase dimer
HEX: high-energy X-ray probe
HR: Heptad Repeat
IC: internal control
Kb: kilobyte
LB: Backward Loop Primer
LED: light-emitting diode
LF: Forward Loop Primer
LFA: Lateral Flow Assay
LFB: Lateral Flow Bioassay
LFD: Lateral Flow Dipstick
LoD: limit of detection
M: membrane glycoprotein
MALDI-TOF-MS: matrix-assisted laser desorption ionization–time of flight mass spectroscopy
MERS-CoV: Middle East Respiratory Syndrome Coronavirus
MG: Malachite Green
mRT-LAMP: Multiplex Reverse Transcription-Loop-Mediated isothermal
mRT-LAMP-LFB: Multiplex Reverse Transcription-Loop Mediated isothermal-
N: Nucleocapsid protein
NAAT: Nucleic Acid Amplification Test
NC: negative control
NEAR: Nicking Enzyme Amplification Reaction
NPA: Negative Agreement
nsp: Nonstructural Protein
NTD: N-Terminal Domain
ORF: Open Reading Form
PaGeR: Palm Germ Reader
PAM: Protospacer Adjacent Motif
PC: positive control
PD: particle diffusometry
Pfu: Proofreading DNA Polymerase
PL-pro: Papain-like protease
PNA: peptide nucleic acid
POCT: Point of Care Testing
pp: polypeptides
PPA: Positive Agreement
PTSs: pregnancy test strips
qSTAR: Selective Temperature Amplification Reaction
RAVI-CRISPR: Rapid Visual-CRISPR
RBD: Receptor Binding Domain
RCA: Rolling Circle Amplification
RdRp: RNA-dependent RNA polymerase
RLU: Relative Light Unit
RNase P: Ribonuclease P
ROX: Carboxy-X-rhodamine
RPA: Recombinase Polymerase Amplification
RT-ddPCR: Real Time-Droplet Digital Polymerase Chain Reaction
RT-LAMP: Reverse Transcription-Loop Mediated Isothermal Amplification
RT-PCR: Reverse Transcription-Polymerase Chain Reaction
S: Spike protein
SAP: Short Amplicon Primer
SARS-CoV-2: Severe Acute Respiratory Syndrome Coronavirus 2
SD: Subdomain
SHERLOCK: Specific High-Sensitivity Enzymatic Reporter Unlocking
SNV: single nucleotide variants spectroscopy
SPEs: screen-printed electrodes
ssRNA: single-stranded RNA
TM: Transmembrane Domain
TMA: transcription-mediated amplification
TMSE: Toehold-Mediated Strand Exchange
TOPSE: True Outcome Predicted through Strip Evaluation
UDG-RT-LAMP: Uracil-DNA-Glycosylase-Reverse Transcription-Loop Mediated
VOC: Variants of Concern
WHO: World Health Organisation
